# *Trypanosoma brucei* multi-aminoacyl-tRNA synthetase complex formation limits promiscuous tRNA proofreading

**DOI:** 10.3389/fmicb.2024.1445687

**Published:** 2024-07-16

**Authors:** Rylan R. Watkins, Anna Vradi, Irina Shulgina, Karin Musier-Forsyth

**Affiliations:** Department of Chemistry and Biochemistry, Center for RNA Biology, Ohio State University, Columbus, OH, United States

**Keywords:** aminoacyl-tRNA synthetases, *trans*-editing, tRNA, *Trypanosoma brucei*, translation, multi-aminoacyl-tRNA synthetase complex

## Abstract

Faithful mRNA decoding depends on the accuracy of aminoacyl-tRNA synthetases (ARSs). Aminoacyl-tRNA proofreading mechanisms have been well-described in bacteria, humans, and plants. However, our knowledge of translational fidelity in protozoans is limited. *Trypanosoma brucei* (*Tb*) is a eukaryotic, protozoan pathogen that causes Human African Trypanosomiasis, a fatal disease if untreated. *Tb* undergoes many physiological changes that are dictated by nutrient availability throughout its insect-mammal lifecycle. In the glucose-deprived insect vector, the tsetse fly, *Tb* use proline to make ATP via mitochondrial respiration. Alanine is one of the major by-products of proline consumption. We hypothesize that the elevated alanine pool challenges *Tb* prolyl-tRNA synthetase (ProRS), an ARS known to misactivate alanine in all three domains of life, resulting in high levels of misaminoacylated Ala-tRNA^Pro^. *Tb* encodes two domains that are members of the INS superfamily of aminoacyl-tRNA deacylases. One homolog is appended to the N-terminus of *Tb* ProRS, and a second is the major domain of multi-aminoacyl-tRNA synthetase complex (MSC)-associated protein 3 (MCP3). Both ProRS and MCP3 are housed in the *Tb* MSC. Here, we purified *Tb* ProRS and MCP3 and observed robust Ala-tRNA^Pro^ deacylation activity from both enzymes *in vitro*. Size-exclusion chromatography multi-angle light scattering used to probe the oligomerization state of MCP3 revealed that although its unique N-terminal extension confers homodimerization in the absence of tRNA, the protein binds to tRNA as a monomer. Kinetic assays showed MCP3 alone has relaxed tRNA specificity and promiscuously hydrolyzes cognate Ala-tRNA^Ala^; this activity is significantly reduced in the presence of *Tb* alanyl-tRNA synthetase, also housed in the MSC. Taken together, our results provide insight into translational fidelity mechanisms in *Tb* and lay the foundation for exploring MSC-associated proteins as novel drug targets.

## 1 Introduction

*Trypanosoma brucei* (*T. brucei*; *Tb*) is a sub-Saharan protozoan species and the cause of Human African Trypanosomiasis (HAT) (Filardy et al., 2018; Jamabo et al., 2023). Historically, drugs targeting *Tb* infection are unsafe and unreliable, as many are toxic to both the parasite and human host, like arsenic based Melarsoprol (Bernhard et al., 2022). A newer, safer therapeutic, Fexinidazole, is now the standard treatment for HAT infections (Bernhard et al., 2022). Despite the success of Fexinidazole, novel therapeutics are needed to ensure patient accessibility and to account for potential incidences of drug resistance (Jamabo et al., 2023).

*T. brucei* assumes two major forms, procyclic and bloodstream, depending on their host (Toh et al., 2021). The procyclic stage of the parasite is present in the midgut of its insect vector, the Tsetse fly (*Glossina*). To advance its lifecycle, procyclic *Tb* migrate to the fly salivary glands, where they differentiate into an intermediary form, epimastigote, which eventually transitions into an infectious metacyclic form (Clayton, 2019; Toh et al., 2021). Bloodstream parasites arise after the tsetse fly transmits metacyclic *Tb* to a mammalian host. Bloodstream *Tb* encode a variant surface glycoprotein coat, which is antigenically varied to escape the innate immune response (Roditi and Liniger, 2002; Silva Pereira et al., 2022). The procyclic parasites use a similar strategy to survive within the fly, but instead encode a glycosylphosphatidylinositol-anchored, glutamate- and proline-rich protein coat (procyclin); previous studies showed that the procyclin coat is necessary for *Tb* colonization of the tsetse midgut (Ruepp et al., 1997; Acosta-Serrano et al., 1999). Bloodstream and procyclic *Tb* acquire energy differently (Smith et al., 2017). Although aerobic, bloodstream parasites proliferate by using glucose to generate ATP via glycolysis in specialized organelles called glycosomes. Glycosomes are absent in the procyclic form, as the midgut tsetse fly is glucose poor. Instead, proline is abundant and is imported into the mitochondrion for ATP production by oxidative phosphorylation. Proline is initially converted to glutamate, which then undergoes transamination with pyruvate to form alanine and ⍺-ketoglutarate (⍺-KG). Alanine is assimilated into the free amino acid pool for use by Tb or excreted from the cell, while ⍺-KG enters the tricarboxylic acid cycle to eventually produce ATP (Mantilla et al., 2017; Marchese et al., 2018; Haindrich et al., 2021).

In the procyclic and bloodstream forms of *T. brucei*, alanine is one of the most abundant amino acids (Williamson and Desowitz, 1961; O'Daly et al., 1983; Creek et al., 2015; Marchese et al., 2018; Johnston et al., 2019) and as a result, may challenge the fidelity of aminoacyl-tRNA synthetases (ARSs), the enzymes responsible for aminoacyl-tRNA (aa-tRNA) synthesis. The aminoacylation reaction occurs in two steps: (1) amino acid activation with ATP and (2) transfer of the aminoacyl-adenylate to the cognate tRNA (Rubio Gomez and Ibba, 2020). Some ARSs mis-select non-cognate amino acids smaller than or structurally similar to the cognate substrate (Kuzmishin Nagy et al., 2020; Jani and Pappachan, 2022). Organisms evolved pre- and post-transfer proofreading mechanisms to maintain fidelity during aminoacylation. Pre-transfer editing mechanisms hydrolyze misactivated amino acids, while post-transfer mechanisms clear mischarged tRNAs in *cis* (before aa-tRNA release) or in *trans* (after release) (Ahel et al., 2003; Ling et al., 2009; Kuzmishin Nagy et al., 2020). *Trans*-editing can be performed by an ARS or by dedicated freestanding proofreading factors.

Prolyl-tRNA synthetases (ProRS) catalyze the aminoacylation of cognate proline to tRNA^Pro^. ProRSs across the three domains of life misactivate alanine and cysteine (Beuning and Musier-Forsyth, 2001; Ahel et al., 2002). Most bacterial ProRSs have an editing domain (INS) inserted between the class II ARS consensus motifs 2 and 3, which serves to hydrolyze Ala-tRNA^Pro^, and encode a *trans*-editing enzyme, YbaK, to clear Cys-tRNA^Pro^ (Beuning and Musier-Forsyth, 2000; Wong et al., 2003; An and Musier-Forsyth, 2004). Organisms lacking an INS domain encode a homologous protein, ProXp-ala, to edit Ala-tRNA^Pro^ in *trans* (Vargas-Rodriguez and Musier-Forsyth, 2013). A bioinformatics sequence similarity network analysis showed that the INS superfamily consists of nine related but distinct protein clusters (Kuzmishin Nagy et al., 2020). Although the INS superfamily members characterized to date have different amino acid and tRNA substrate specificities, all use a highly conserved lysine and Gly-XXX-Pro (GXXXP) loop for aa-tRNA stabilization during hydrolysis (Bartholow et al., 2014; Kuzmishin Nagy et al., 2020; Jani and Pappachan, 2022). The INS superfamily members characterized to date include ProXp-ala (Ala-tRNA^Pro^ deacylase) (Ahel et al., 2003; Vargas-Rodriguez and Musier-Forsyth, 2013; Danhart et al., 2017; Vargas-Rodriguez et al., 2020; Byun et al., 2022; Ma et al., 2023), YbaK (Cys-tRNA^Pro^ deacylase) (An and Musier-Forsyth, 2004, 2005; Kumar et al., 2013; Das et al., 2014; Chen et al., 2019), ProXp-x (Ala- and ⍺-aminobutyrate-tRNA^Pro^ deacylase) (Bacusmo et al., 2018), and YeaK (Ser- and Thr-tRNA deacylase) (Liu et al., 2015; McMurry and Chang, 2017; Bacusmo et al., 2018). Three additional clusters of the INS superfamily (designated ProXp-7,-8,-9) remain uncharacterized—one of the three clusters, ProXp-7, includes *Tb* multi-aminoacyl-tRNA synthetase complex (MSC)-associated protein 3 (MCP3), reported to be a core member of the *Tb* MSC (Cestari et al., 2013; Kuzmishin Nagy et al., 2020).

Our understanding of aminoacylation and fidelity mechanisms in *Tb* is far from complete. Due to the metabolic strategy of procyclic stage parasites, we expect the exaggerated alanine pool causes elevated alanine mischarging by *Tb* ProRS. We hypothesize that *T. brucei* evolved robust Ala-tRNA^Pro^ proofreading strategies to maintain translational fidelity. In addition to MCP3, an INS superfamily member of unknown function, *Tb* ProRS encodes an N-terminal INS-like domain previously reported to be present in a subset of lower eukaryotes and shown to be an Ala-tRNA^Pro^ deacylase *in vitro* in another protozoan parasite, *Plasmodium falciparum* (Ahel et al., 2003). Both *Tb* ProRS and MCP3 are members of the MSC, along with five other ARSs—methionyl-tRNA synthetase (MetRS), glutaminyl-tRNA synthetase (GlnRS), aspartyl-tRNA synthetase (AspRS), tryptophanyl-tRNA synthetase (TrpRS), and alanyl-tRNA synthetase (AlaRS)—and two other tRNA-binding/MSC-associated proteins (MCP1 and MCP2) (Cestari et al., 2013).

In this study, we recombinantly expressed and purified *Tb* MCP3 and full-length (FL) ProRS from *Escherichia coli* for the first time. Domain deletion mutants were also investigated. We determined that both *Tb* MCP3 and FL ProRS are robust Ala-tRNA^Pro^ deacylases *in vitro*. Enhanced deacylation was observed with FL ProRS in comparison to the activity of the ProRS INS-like N-terminal domain (NTD) alone, suggesting a potential evolutionary advantage of the fusion. Size-exclusion chromatography followed by multi-angle light scattering (SEC-MALS) analysis revealed the NTD of MCP3 is essential for homodimerization, but kinetic assays and isothermal titration calorimetry (ITC) analysis showed that the NTD only marginally contributes to catalysis and tRNA binding affinity. We investigated the tRNA specificity of MCP3 and observed poor discrimination, as Ala-tRNA^Ala^ was robustly hydrolyzed. MCP3 activity was tested in the context of other *Tb* MSC members (AlaRS and ProRS); *Tb* AlaRS effectively protected Ala-tRNA^Ala^ from *trans* editing by MCP3. This work highlights the robust proofreading capability of two *Tb* proteins and the role of the MSC in keeping the promiscuous activity of MCP3 in check. We speculate MCP3 is advantageous to parasite fitness, as *Tb* likely requires robust translational quality control mechanisms to counteract the physiological demands of its lifecycle. The absence of MCP3 from the human genome suggests that it could be a valuable drug target in the development of therapeutics against HAT and related protozoan diseases.

## 2 Materials and methods

### 2.1 Multiple sequence alignments and structural modeling

DNA and amino acid sequences for *Tb* MCP3 (Tb927.10.1250) and *Tb* ProRS (Tb927.10.12890) were obtained from the Kinetoplastid Informatics Resource, TriTrypDB. All other amino acid sequences of INS superfamily members were collected from UniProt. Multiple sequence alignments were constructed by first performing a structural alignment using the pairwise alignment tool available from the Protein Data Bank (PDB). Structural alignments of an AlphaFold2 (Jumper et al., 2021) prediction of *Tb* MCP3, *Hi* YbaK (PDB: 1DBX), *Cc* ProXp-ala (PDB:5VXB), and an AlphaFold2 prediction of *At* ProXp-ala were used as constraints in a multiple-sequence alignment using the MAFFT 7 alignment tool (Katoh et al., 2017). EMBL Clustal Omega was used to present the alignment results (Madeira et al., 2022).

### 2.2 Plasmid DNA and site-directed mutagenesis

Sequences of all proteins were codon optimized for expression in *E. coli*. Genes encoding *Tb* MCP3 and *Tb* ProRS were commercially synthesized and cloned into a pET15b vector by Azenta Technologies. The gene for *Tb* AlaRS was synthesized and cloned into a pGS21 vector by GenScript. Coding sequences were taken from TriTrypDB with exception of *Tb* ProRS, which was taken from UniProt, as the coding region annotated on TriTrypDB was lacking the mitochondrial targeting sequence (MTS). Genes encoding tRNAs of interest with an added upstream T7 promoter were commercially synthesized and cloned into pUC vectors by Azenta Technologies or Integrated DNA Technologies (IDT). Mutagenesis of tRNA genes for making *Tb* tRNA^Pro^ acceptor stem mutants, deletion of the NTD of the *Tb* MCP3 gene, and deletion of the MTS and ProXp-ala domain from *Tb* ProRS was performed using site-directed ligase independent mutagenesis (SLIM) according to published protocols (Chiu et al., 2004). All DNA primers were purchased from IDT.

### 2.3 Protein purification

pET15b *Tb* MCP3 (N-His_6_), pET15b *Tb* ProXp-ala (ΔProRS; N-His_6_), pET15b *Tb* ΔProXp-ala ProRS (ΔMTS; N-His_6_) and pGS21a *Tb* AlaRS (N-His_6_-GST) were electroporated into Rosetta (DE3) *E. coli* (Novagen) for expression. pET15b *Tb* ProRS (ΔMTS; N-His_6_) was transformed into New England Biolabs (NEB) T7 Express SHuffle pLysS. Rosetta DE3 *E. coli* expressing the protein of interest was grown in LB media (0.5–2 L), or terrific broth for *Tb* AlaRS, with antibiotics (100 μg/mL ampicillin, 30 μg/mL chloramphenicol) at 37°C, 220 RPM until the OD_600_ was between 0.5–0.8. Flasks were chilled on ice for 15 min prior to addition of isopropyl-β-D-1-thiogalactopyranoside (IPTG) to a final concentration of 0.1 mM. Proteins were induced overnight (16–18 h) at 18°C. All proteins expressed in Rosetta were isolated similarly, with the difference being composition of the purification buffer (isolation buffer) (see Supplementary Table S1). Briefly, for protein preparation, induced cells were pelleted at 6000 × g for 10 min at 4°C and then resuspended in pre-chilled isolation buffer at a ratio of 10 mL per gram of cell pellet. One cOmplete EDTA-free protease inhibitor tablet (Roche) and 50 mg of lysozyme powder (Sigma) was added per 50 mL of cell suspension. Cells were incubated for 30 min on ice with lysozyme before a sonication cycle of 15 s on, 45 s off, at 50% amplitude for a total of 8 pulses. Total lysate was clarified at 20,000 × g for 30 min, 4°C. The soluble fraction was loaded onto a gravity flow column packed with EDTA-compatible high-capacity nickel resin (ThermoFisher) equilibrated with isolation buffer. Proteins were eluted with an imidazole step gradient (20–500 mM) and fractions analyzed by denaturing polyacrylamide gel electrophoresis (SDS-PAGE). Peak protein fractions were pooled, then concentrated and buffer exchanged three times with storage buffer (Supplementary Table S1) by Amicon Ultra molecular-weight cutoff (MWCO) centrifugal filters (Sigma).

Full-length *Tb* ProRS (ΔMTS) was prepared by growing the transformed SHuffle pLysS *E. coli* cells in 3 L of enriched terrific broth (20 g bacto tryptone, 24 g bacto yeast extract, 10 mM MgCl_2_, 25 mM KP_i_ pH 7.0, 0.4% v/v glycerol) with 100 μg/mL ampicillin. Cultures were grown to OD_600_ of 0.5–0.8 at 37°C and then chilled on ice for 15 min. Cultures were supplemented with 100 μM ZnCl_2_, refreshed with an additional 50 μg/mL ampicillin, and then induced with 0.25 mM IPTG overnight at 16°C. Induced cells were pelleted at 6000 × g for 10 min, 4°C, and then resuspended in 30 mL of isolation buffer (Supplementary Table S1) supplemented with cOmplete EDTA-free protease inhibitor tablet (Roche), 1% v/v IGEPAL CA-630 (ThermoFisher), and 20% D-trehalose (Research Products International). Cells were lysed by sonication: 5 s on, 25 s off, 70% amplitude for a total of 18 pulses. Sonicated cell suspension was diluted to 100 mL with isolation buffer (see Supplementary Table S1), stirred for an additional 10 min on ice, and clarified at 20,000 × g for 30 min, 4°C. Soluble protein was bound to a nickel affinity resin pre-equilibrated with isolation buffer via a batch method wherein 2.5 mL of high-capacity nickel resin was incubated with 100 mL of clarified lysate for 20 min at 4°C on a tube rotator. Protein-bound resin was recovered by centrifugation at 2000 × g for 10 min at 4°C, resuspended in 15 mL of isolation buffer and then transferred to a gravity flow column for washes and elution. One 50 mL wash with isolation buffer and two 10 mL high-salt washes (isolation buffer +0.5 M (NH_4_)_2_SO_4_ + 15 mM imidazole) were performed before eluting ProRS with a 40–640 mM imidazole step gradient. Fractions containing ProRS were identified by SDS-PAGE, concentrated to 0.5 mL using a 50 kDa MWCO Amicon Ultra centrifugal filters (Sigma) and immediately loaded onto to a Superdex 200 Increase 10/300 GL gel filtration column (Cytiva) pre-equilibrated with 50 mM MOPS pH 7.8, 1.5 M NaCl, 10 mM DTT, 5% glycerol. Gel filtration fractions containing FL ProRS were identified by SDS-PAGE, buffer exchanged three times using 50 kDa MWCO Amicon Ultra centrifugal filters (Sigma) with 2X storage buffer (Supplementary Table S1) and then mixed 1:1 with 90% v/v glycerol before storage at −20°C.

Protein concentration and nucleic acid contamination was estimated via absorbance at 280 nm and 260 nm, respectively, using a nanodrop spectrophotometer and molar extinction coefficients that were predicted with Expasy ProtParam. Calculated concentrations were validated on an analytical SDS-PAGE against a BSA standard. Activities of purified ARSs were determined by active-site titration as described previously (Fersht et al., 1975; Francklyn et al., 2008).

### 2.4 *In vitro* transcription of tRNA

Templates for *in vitro* transcription by T7 RNA polymerase were prepared by PCR using Phusion high-fidelity DNA polymerase (NEB). DNA amplicons were purified using a PCR clean-up kit (Qiagen) and 10–20 μg was added to a 1 mL transcription reaction containing 80 mM HEPES-K, pH 8.0, 30 mM Mg(OAc)_2_, 10 mM DTT (Research Products International), 5 mM spermidine, 0.01% Triton-X-100, 2% v/v PEG8000, 4 mM rNTPs (ThermoFisher), 5 U inorganic yeast pyrophosphatase (Sigma), and recombinant P266L T7 RNA polymerase (Ramírez-Tapia and Martin, 2012; Chillón et al., 2015). Transcriptions were incubated at 37°C for 4 h or overnight at 25°C and terminated with 50 mM EDTA, pH 8.0. tRNAs were purified by Urea-PAGE and gel elution according to published protocols (Petrov et al., 2013), or by anion exchange chromatography (Koubek et al., 2013; Petrov et al., 2013). For anion exchange chromatography, tRNAs were loaded onto a HiTrap Q-HP (Cytiva) column connected to an AKTA Pure FPLC system (Cytiva), equilibrated with 20 mM Tris–HCl, pH 7.5, 150 mM NaCl. Bound tRNAs were eluted from the column using a 40-column volume linear gradient (300–1,000 mM NaCl) with a buffer consisting of 20 mM Tris–HCl pH 7.5. FPLC fractions containing the tRNA were identified by Urea-PAGE, pooled, concentrated by centrifugal ultrafiltration with 3 kDa MWCO Amicon Ultra centrifugal filters (Sigma), and ethanol precipitated. tRNAs were resuspend in MilliQ-H_2_O before determining the concentration by A_260_ on a Nano-drop spectrophotometer (*ε*_260_ = 604,000 M^−1^ cm^−1^).

### 2.5 Size-exclusion chromatography coupled to multi-angle light scattering

WT and ΔN MCP3 (300 μg each) were diluted into mobile phase buffer containing 30 mM Tris–HCl pH 8.0, 100 mM NaCl, 5 mM MgCl_2_, and 2 mM DTT to a final concentration of 3 mg/mL in 100 μL. Samples were incubated at 27°C for 1 h before all 300 μg of protein was loaded onto a pre-equilibrated Superdex 200 Increase 10/300 GL gel filtration column (Cytiva) connected to an AKTA Pure FPLC system equipped with Dawn 8+ MALS and T-Rex refractive index detectors (Wyatt). Protein separations and data collection occurred at a flow rate of 0.5 mL/min for a total of 50 min. Molar mass was determined by analyzing MALS data using ASTRA v7.1.4 software according to manufacturer’s guidelines. Folded tRNA^Pro^ was diluted to 1.5 mg/mL in 100 μL of mobile phase buffer. For tRNA binding studies, 60 μM of folded tRNA^Pro^ was incubated with 150 μM of WT or ΔN MCP3 in a final volume of 100 μL at 27°C for 1 h. The entire sample was loaded onto the gel filtration column for MALS analysis.

### 2.6 Aminoacyl-tRNA substrate preparation

*In vitro* transcribed tRNAs (100 pmol) were 3′-end labeled with ^32^P via nucleotide exchange of the terminal adenosine (A_76_) using [^32^P]-ATP and *E. coli* nucleotidyltransferase according to published protocols (Ledoux and Uhlenbeck, 2008). [^32^P]-tRNAs were ethanol precipitated in the presence of 30 μg GlycoBlue (ThermoFisher) and the pellet was resuspended with ~3,000 pmol of non-radiolabeled tRNA in 10 mM HEPES-KOH, pH 7.0. tRNAs were renatured by heating at 80°C for 2 min, 60°C for 2 min, and addition of 10 mM MgCl_2_ followed by a slow cool to room temperature for 5 min. Folded [^32^P]-tRNA^Pro/Ala^ (5–10 μM) was (mis)charged by incubation at 37°C with 1 μM of cognate *Tb* ARS, saturating amino acid, and buffer (50 mM HEPES-K pH 7.5, 20 mM KCl, 10 mM MgCl_2_, 0.1 mg/mL bovine serum albumin, 4 mM ATP, 2 mM TCEP, 5 U yeast inorganic pyrophosphatase) for 30 min. For mischarging tRNA^Pro^ with alanine, a ΔProXp-ala Tb ProRS mutant was used. Charging was terminated by extraction with acidic phenol:chloroform, pH 4.5 (Ambion) and aa-tRNAs were recovered by ethanol precipitation.

For flexizyme-catalyzed charging (G1C:C72G, C73A and C73A tRNA^Pro^ mutants and tRNA^Leu,Thr,Trp^), 10 μM [^32^P]-tRNA was mixed with equimolar amounts of dinitroflexizyme (dFx) (Murakami et al., 2006; Goto et al., 2011) in 100 mM HEPES-K, pH 7.5, then renatured by heating at 95°C for 2 min and 60°C for 2 min followed by addition of 0.1 M of MgCl_2_ and 0.1 M KCl. The RNAs were cooled to RT for 5 min and then incubated on ice. dFx charging was initiated by addition of 5 mM dinitrobenzylester-activated amino acid (Ala-, Gly-, Ser-, or Leu-DBE), prepared as described (Murakami et al., 2006; Goto et al., 2008). After incubation on ice for 2 h (Ala, Gly, Ser) or 24 h (Leu), charging was quenched with 0.2 M NaOAc, pH 5.1 and aa-tRNAs were recovered by ethanol precipitation. Aa-tRNAs were resuspended in 5 mM NaOAc, pH 5.1 and stored at −80°C. The concentration of aa-tRNAs was determined by polyethylenimine-celluose thin layer chromatography (TLC) as described previously (Ledoux and Uhlenbeck, 2008; Byun et al., 2022). TLC plates were exposed to phosphorimaging cassettes (Cytiva) for ~16 h and then imaged with a Typhoon RGB phosphorimager (Cytiva). ImageQuant was used for densitometric analysis of TLC plate images.

### 2.7 Deacylation assays

For single-turnover assays, a 2X enzyme mix and a 2X aa-tRNA mix were prepared separately: enzymes were diluted to 1 μM with 2X reaction buffer (100 mM Tris–HCl pH 7.5, 20 mM MgCl_2_, 60 mM KCl, 4 mM DTT, 0.2 mg/mL BSA) and aa-tRNA was diluted to 100 nM with Milli-Q water. Both 2X mixes were incubated at the desired reaction temperature (13.5°C or 27°C) for at least 2 min before initiating the reaction by addition of 2X enzyme into 2X aa-tRNA. At desired time points, 2 μL were quenched into 6 μL of 250 mM NaOAc, pH 5.1, 280 mM NaCl, 4.5 mM ZnSO_4_, 0.5 U/ μL of S1 nuclease (Promega). Quenched time points were incubated at room temperature for 30 min, before analyzing digestion products by TLC and imaging as described above. ImageQuant (Cytiva) was used to obtain the densitometric ratio of charged to uncharged tRNA, which was then plotted against time and fit to a single-exponential decay equation with GraphPad Prism to obtained rate constants (*k*_obs_). Multiple-turnover assays were conducted similarly, except with 20 nM enzyme and 200 nM aa-tRNA. Results from multiple-turnover assays were fit to a linear regression to obtain initial velocities. In some assays, MCP3 was pre-incubated with an equimolar amount (1 μM, 2X final concentration) of *Tb* AlaRS, ProRS, or *Hs* LysRS in a buffer containing 100 mM Tris–HCl pH 7.5, 20 mM MgCl_2_, 60 mM KCl, 4 mM DTT, 0.2 mg/mL BSA for 20 min at 27°C before initiating the deacylation assay by addition of equal volume of 100 nM Ala-tRNA^Ala^ prepared in Milli-Q water.

### 2.8 Isothermal titration calorimetry

Prior to binding assays, concentrated tRNA stocks (~500 μM) were renatured in 20 mM MOPS-KOH, pH 7.8 and 100 mM NaCl by heating at 80°C for 3 min, 60°C for 2 min, followed by addition of 10 mM MgCl_2_ and slow cool to room temperature. WT and ΔN MCP3 were diluted to 50 μM in ITC buffer A (20 mM MOPS pH 7.8, 100 mM NaCl, 2 mM dithioerythritol (DTE), 1 mM MgCl_2_). Folded tRNAs and diluted proteins were loaded into separate 3 kDa MWCO Slide-A-Lyzer dialysis cassettes (ThermoFisher) and then co-dialyzed against 3 L of ITC buffer A overnight (12–18 h). Dialyzed samples were passed through a 13 mm, 0.2 μm Whatman syringe filter (Cytiva) prior to concentration determination by UV absorbance using a Nano-drop spectrophotometer (Thermofisher) and *ε*_280_ of WT (*ε*_280_ = 32,430 M^−1^ cm^−1^) and ΔN MCP3 (*ε*_280_ = 30,940 M^−1^ cm^−1^). Protein and tRNA samples were diluted to 30 μM and 300 μM with ITC buffer A, respectively, and equilibrated to assay temperature (25°C) on a heat block for 5 min. A MicroCal PEAQ-ITC (Malvern Panalytical) was used for data collection. Protein sample was loaded into the titration cell pre-equilibrated with ITC buffer A and tRNA was loaded into the titration syringe. The titration was performed in high-feedback mode by the addition of 19, 1.3 μL injections separated by 180 s with a stirring speed of 620 rpm. Data was analyzed with the MicroCal PEAQ-ITC software according to manufacturer’s guidelines.

For MCP3 dimer dissociation experiments, a concentrated stock of WT MCP3 (~500 μM) was exchanged into ITC buffer B (50 mM Glycylglycine, pH 8.0, 300 mM NaCl, 2 mM DTE) using a 10 K MWCO Amicon ultrafiltration centrifugal membrane (Sigma), concentrated to 1 mL, and then loaded into a 10 kDa MWCO Slide-A-lyzer dialysis cassette (ThermoFisher). WT MCP3 was dialyzed in against 2 L of ITC buffer B overnight (12–16 h). The dialyzed sample was filtered, and concentration determined as described above. WT MCP3 was diluted to 250 μM, loaded into the titration syringe, and injected into the ITC cell filled with ITC buffer B. Data was collected across 13, 1 μL injections at 25°C with a stirring speed of 600 rpm.

## 3 Results

### 3.1 Sequence and structure analysis of *Tb* MCP3 and ProRS

**Figure 1:**
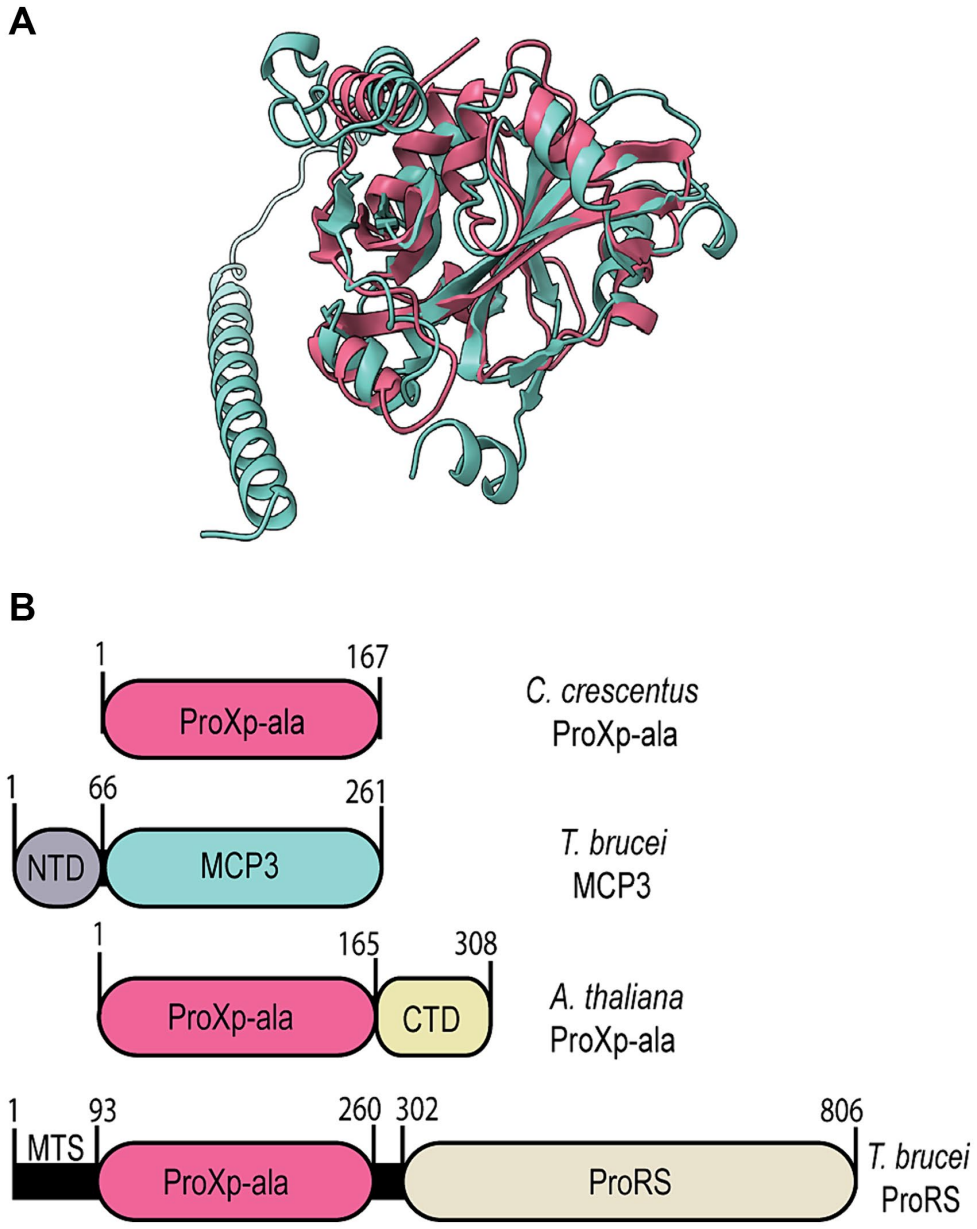
Structural comparison of MCP3 and ProXp-ala domains. **(A)** AlphaFold2 structure prediction of *Tb* MCP3 (teal) overlayed on an X-ray crystal structure of *Cc* ProXp-ala (Red; PDB: 5VXB). **(B)** Domain architecture of *Tb* MCP3 and ProXp-ala factors.

**Figure 2:**
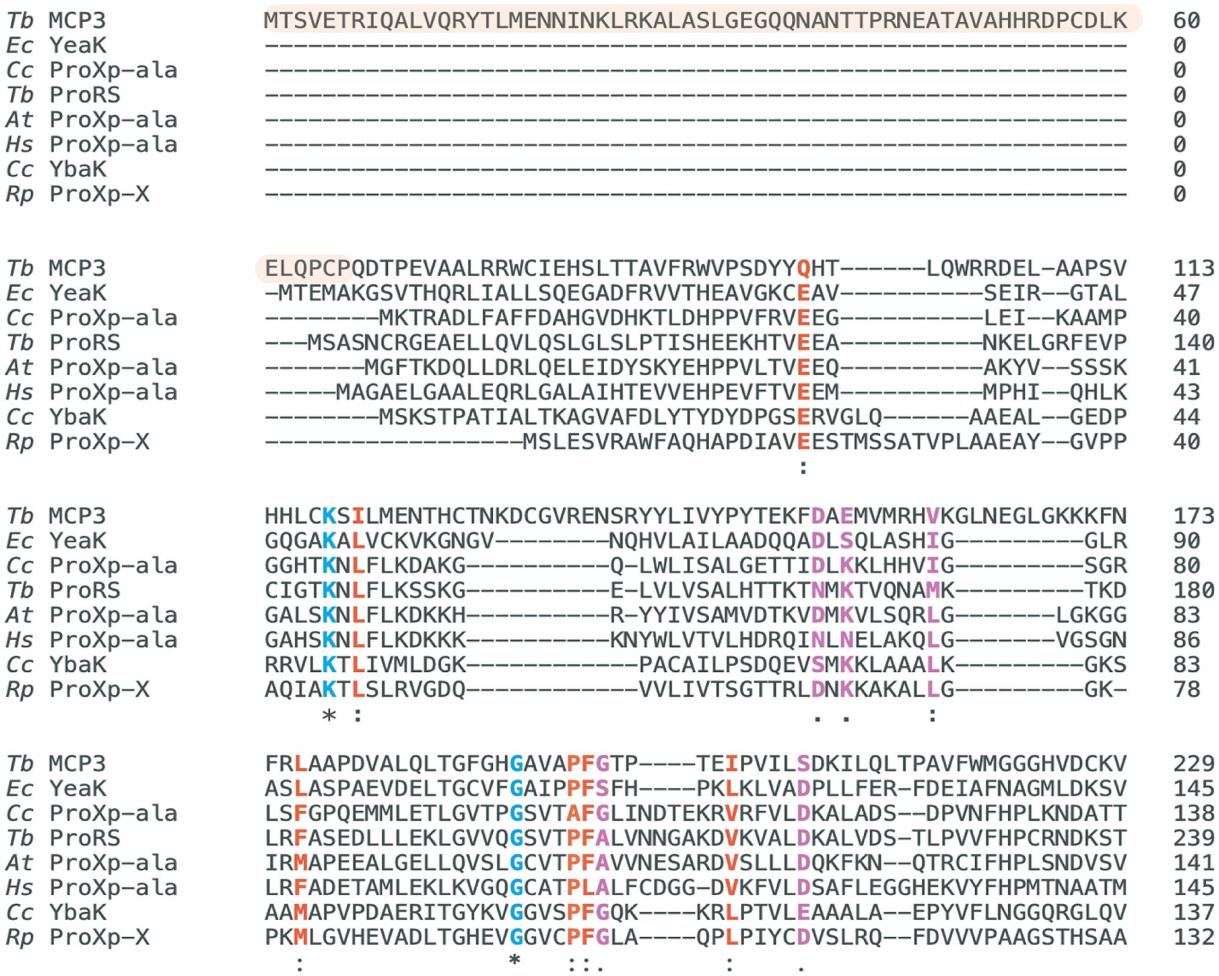
Multiple-sequence alignment of INS superfamily members. Highlighted in blue are strictly conserved catalytic residues (*), in red are highly similar residues (:), and in purple are moderately similar residues (.). The MCP3 sequence highlighted in orange is the unique N-terminal domain. Residue conservation determined and scored with the BLOSUM62 matrix via the MAFFT7 alignment tool. Clustal Omega was used to generate the alignment figure. *Tb* ProRS sequence represents the N-terminal ProXp-ala domain (94–260). The alignment presented represents the regions with the highest similarity—the entire sequence of each protein is not shown. *Tb*, *Trypanosoma brucei*; *At*, *Arabidopsis thaliana*; *Cc*, *Caulobacter crescentus*; *Rp*, *Rhodopseudomonas paulustris*; *Ec*, *Escherichia coli*; *Hs*, *Homo sapiens*.

Bioinformatic analyses revealed *Tb* MCP3 is not found in the human genome and is solely encoded by eukaryotes of the fungi, plant, and protozoan kingdoms (Kuzmishin Nagy et al., 2020). We performed a direct sequence alignment of *Tb* MCP3 using the EMBL Clustal Omega tool with sequences of other INS-like members of known function to locate the conserved lysine and GXXXP catalytic motifs. Direct sequence alignment did not initially identify any highly conserved residues. Next, we used the pairwise alignment tool from the Protein Data Bank (PDB) to generate a structure-based sequence alignment between an AlphaFold2 model of MCP3 and published crystal structures of *Caulobacter crescentus (Cc)* ProXp-ala (PDB: 5VXB) and *Haemophilus influenzae* YbaK (1DBX). An overlay of the *Cc* ProXp-ala structure and the AlphaFold2 model of *Tb* MCP3 is shown in Figure 1A. The alignments generated with the PDB tool were used as constraints to generate the multiple-sequence alignment shown in Figure 2 using the MAFFT 7 software with BLOSUM62 scoring parameters. The structure-based sequence alignment revealed the predicted catalytic domain of MCP3 contains a strictly conserved lysine (K118) and conserved GXXXP (G191-P195) loop. Despite this, MCP3 only has ~12–16% sequence similarity to other INS domain homologs—the highest similarity (~16%) is to the NTD of *Tb* ProRS and to *Rhodopseudomonas paulustris* ProXp-x. Highly conserved amino acids in the ProXp-ala *trans*-editing domain family that were previously shown to play a role in binding and catalysis (e.g., *Cc* ProXp-ala numbering: H23, K50, R80, H130 (Danhart et al., 2017; Ma et al., 2023)) are not conserved in the MCP3 family.

The AlphaFold2 structure prediction of MCP3 depicts a catalytic domain (residues 67–261) with a fold highly similar to the X-ray crystal structure of *Cc* ProXp-ala (Figure 1A), plus a unique ⍺-helical NTD (residues 1–66) that is reminiscent of the distinct ⍺-helical C-terminal extension of *Arabidopsis thaliana* (*At*) ProXp-ala (Figure 1B) (Byun et al., 2022). A homology search using NCBI BLAST of the MCP3 NTD did not provide any significant hits. However, a BLAST search of the entire *Tb* MCP3 amino acid sequence and a subsequent multiple-sequence alignment of MCP3-like proteins from fungi, plants, and other protists revealed that most of these factors have an N-terminal extension and a catalytic domain containing numerous strictly conserved residues in addition to the critical lysine and GXXXP of the INS superfamily (Supplementary Figure S1). Some other protists that encode MCP3, like *Trypanosoma cruzi* and *Leishmania*, are also human pathogens (Bhattacharya et al., 2020).

Bioinformatic analysis of *Tb* ProRS revealed that the INS-like NTD (residues 94–260) belongs to the ProXp-ala cluster of the INS superfamily (Figure 1B). The class II ARS consensus motifs and eukaryote-specific C-terminal zinc-binding domain are highly similar in sequence and structure to the ProRS domain of *Homo sapiens* EPRS (Kuzmishin Nagy et al., 2020; Parrot et al., 2021; Vasu et al., 2021). The aminoacylation catalytic domain of *Tb* ProRS is separated from the appended ProXp-ala by a disordered linker (261–302). N-terminal to the ProXp-ala domain is a 93 amino acid region that AlphaFold2 predicts to be highly disordered—this sequence is predicted to be a mitochondrial-targeting sequence (MTS) (Parrot et al., 2021). In plants, bacteria, and higher eukaryotes (metazoans), ProXp-ala domains are freestanding and edit mischarged Ala-tRNA^Pro^ in *trans*. A BLAST search of *Tb* ProRS confirmed previous reports that only protozoans and fungi encode a ProXp-ala-ProRS fusion protein (Ahel et al., 2003; Gowri et al., 2012; Parrot et al., 2021). Notably, many of the protists that encode a ProRS catalytic core with an appended ProXp-ala domain are human pathogens (i.e., *P. falciparum, Leishmania*).

### 3.2 *Tb* MCP3 uses a size-exclusion based mechanism of deacylation and is more promiscuous than *Tb* ProRS

To characterize the deacylation activities of *Tb* MCP3 and ProRS, we recombinantly expressed and purified both proteins as well as freestanding *Tb* ProXp-ala (lacking the MTS and ProRS catalytic core) in *E. coli*. Initial purification attempts resulted in high yields of pure MCP3 and *Tb* ProXp-ala (~30 mg per L of culture) but isolation of soluble *Tb* ProRS was unsuccessful. We deleted the MTS (ΔMTS), expressed the new construct in a Rosetta (DE3) *E. coli* cell line, but observed little change in solubility. Analysis of the amino acid sequence of *Tb* ProRS identified 19 cysteine residues—we postulated ProRS was insoluble due to incorrect disulfide bond formation. We next overexpressed ΔMTS *Tb* ProRS using *E. coli* SHuffle LysY cells (New England Biolabs), which encode disulfide bond isomerases to assist in protein folding. Using this strategy, soluble *Tb* ProRS was recovered in low yields (~1 mg per 3 L of culture). Since the MTS sequence is not present in cytosolic *Tb* ProRS and gets removed from the mitochondrial isoform, we refer to the ΔMTS ProRS as full-length (FL).

**Figure 3:**
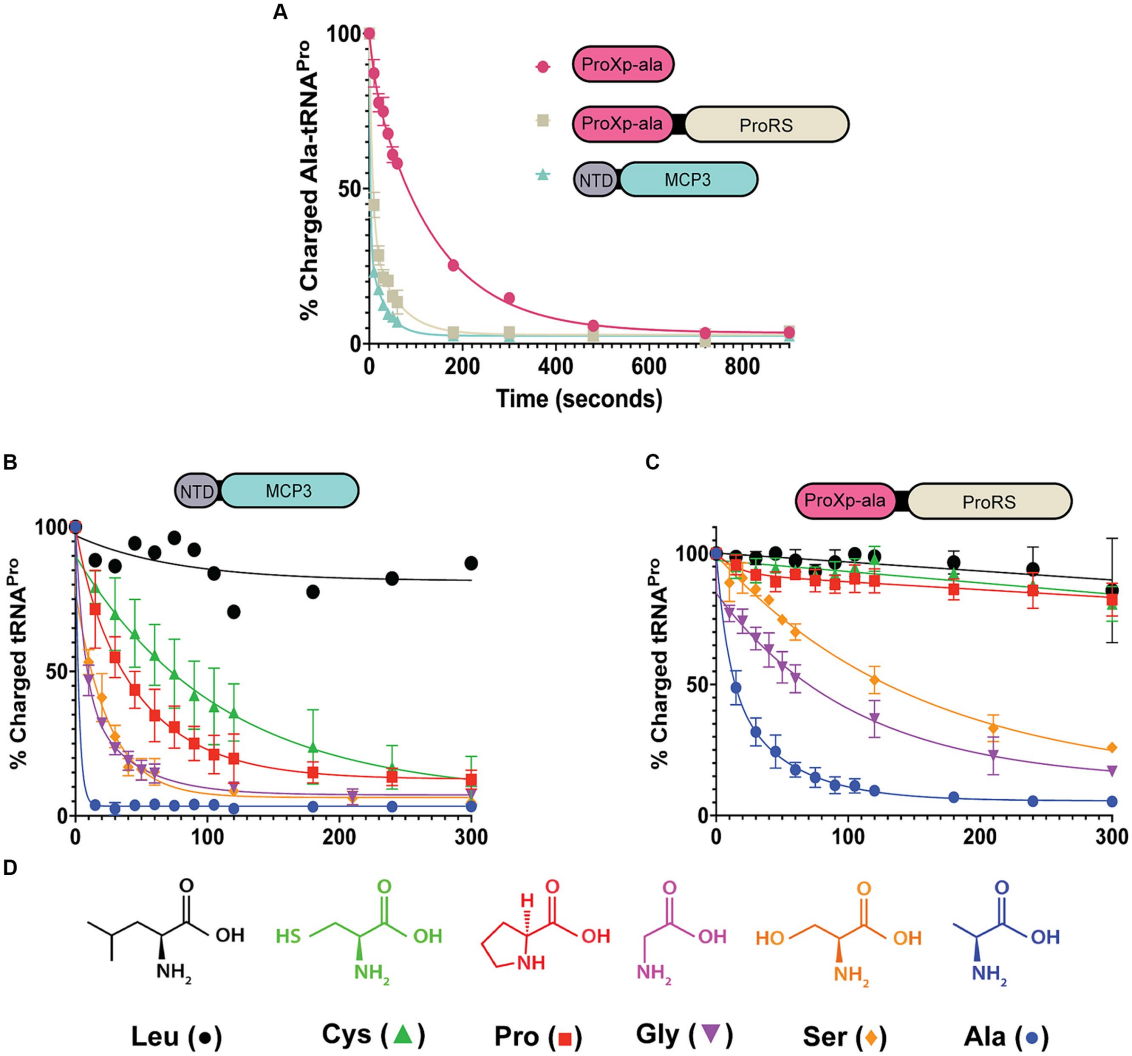
Amino acid specificity of MCP3 and full-length ProRS. Single-turnover kinetics conducted at 27°C with 50 nM aa-tRNA^Pro^ and 500 nM enzyme. **(A)** Comparison of Ala-tRNA^Pro^ deacylase activity of MCP3, ProXp-ala, and FL ProRS. **(B)** Amino acid specificity of MCP3 (Leu; *n* = 1). **(C)** Amino acid specificity of full-length ProRS. Error bars in panels **(A–C)** represent an average of three independent trials. **(D)** Amino acids used in the deacylation assays. Colors and symbols correspond to the data shown in panels **(B,C)**. Rate constants are summarized in Tables 1, 2.

To test the deacylation activity of *Tb* MCP3, FL *Tb* ProRS, and freestanding *Tb* ProXp-ala, we mischarged [^32^P]-labeled *in vitro* transcribed (IVT) *Tb* tRNA^Pro^ with alanine. We observed robust Ala-tRNA^Pro^ deacylation by MCP3 under single-turnover conditions (Figure 3A). The single-turnover rate constant (*k*_obs_) of FL ProRS was ~7-fold slower than the estimated *k*_obs_ of MCP3 (Table 1). Freestanding *Tb* ProXp-ala was ~5-fold slower than FL ProRS, which indicates the ProRS domain promotes Ala-tRNA^Pro^ editing.

**Table 1:** Single-turnover rate constants for deacylation of Ala-tRNA^Pro^ by MCP3, FL ProRS, and the ProXp-ala domain of Tb ProRS.

Enzyme	*k*_obs_ (sec^−1^)*	Fold-Change^†^
MCP3	0.329 ± 0.055	–
FL ProRS	0.046 ± 0.010	7
ProXp-ala	0.009 ± 0.001	37

*k_obs_ values determined using 500 nM enzyme and 50 nM Ala-tRNA^Pro^ at 27°C. Standard deviations represent the average of three independent trials. †Fold-change is based on MCP3 activity.

**Table 2:** Single-turnover rate constants for deacylation of tRNA^Pro^ mischarged with various amino acids.

aa-tRNA^Pro^	MCP3 *k*_obs_ (sec^−1^)*	Fold-Change^†^	FL ProRS *k*_obs_ (sec^−1^)*	Fold-Change^†^
Ala	0.329 ± 0.055	–	0.046 ± 0.010	–
Pro	0.024 ± 0.007	14	ND	>30
Cys	0.011 ± 0.004	30	ND	>30
Gly	0.074 ± 0.005	4.5	0.013 ± 0.003	3.5
Ser	0.055 ± 0.011	6.0	0.006 ± 0.001	7.7
Leu	ND	>30	ND	>30

*k_obs_ values determined using 500 nM enzyme and 50 nM Ala-tRNA^Pro^ at 27°C. Standard deviations represent the average of three independent trials. †Fold-change is relative to Ala-tRNA^Pro^ deacylation activity. ND, not detected.

We next prepared mis-charged tRNAs using amino acids of various sizes and polarities (aa-tRNA^Pro^) (Figure 3D). Due to the structural similarity of *Tb* MCP3 to *Cc* ProXp-ala, we hypothesized MCP3 would display preference for Ala-tRNA^Pro^ and discriminate against larger amino acids. MCP3 and FL ProRS displayed similar aa-tRNA^Pro^ discrimination; both enzymes moderately hydrolyzed Ser- and Gly-tRNA^Pro^ and are unable to edit Leu-tRNA^Pro^ (Figures 3B,C and Table 2). Unlike FL ProRS, *Tb* MCP3 deacylated Pro- and Cys-tRNA^Pro^, which suggests the active site of MCP3 is more promiscuous than the ProXp-ala catalytic pocket (Figure 3B and Table 2).

### 3.3 NTD of MCP3 is essential for homodimerization and is not critical for catalysis or tRNA binding

**Figure 4:**
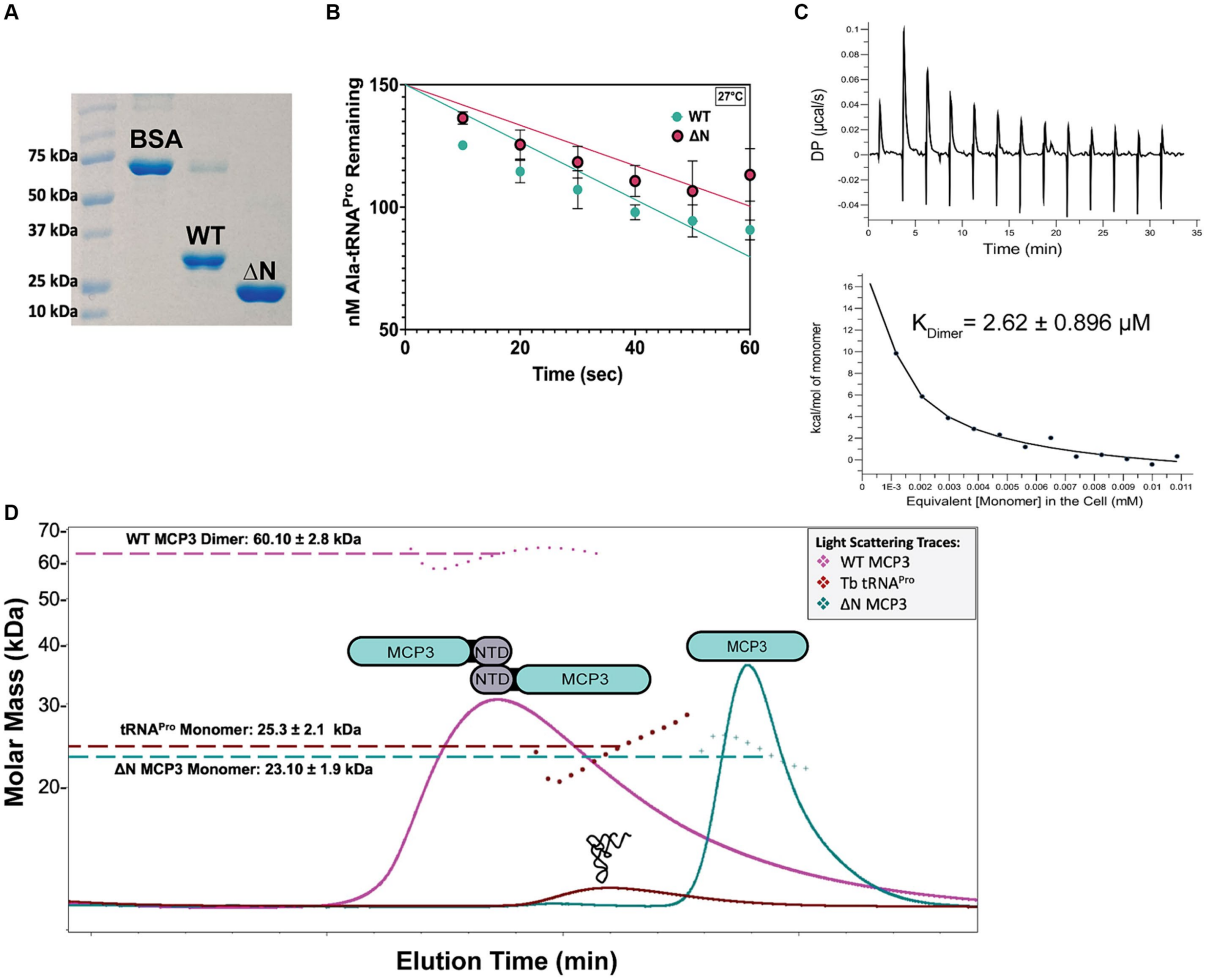
Role of MCP3 NTD in catalysis and homodimerization. **(A)** 12% SDS-PAGE analysis of 3 μg WT and ΔN MCP3 compared to a 3 μg BSA standard. **(B)** Deacylation of Ala-tRNA^Pro^ by WT and ΔN MCP3 at 27°C under multiple-turnover conditions (20 nM protein, 200 nM Ala-tRNA^Pro^). **(C)** Representative ITC results of WT MCP3 homodimer dissociation performed at 25°C as described in methods. Reported K_Dimer_ represents an average of three trials with standard deviation indicated. **(D)** SEC-MALS analysis of WT (31 kDa, pink trace) and ΔN MCP3 (23 kDa, teal trace), and tRNA^Pro^ alone (25 kDa, brown trace). The calculated molar masses represent an average of three independent trials with standard deviation indicated.

Our initial kinetic investigation of MCP3 revealed that it is the most robust Ala-tRNA^Pro^ deacylase within the INS superfamily characterized to date. We previously observed that the C-terminal domain of *At* ProXp-ala improved its catalytic efficiency (~60-fold) and allowed for homodimerization (Byun et al., 2022). To test whether the NTD of MCP3 played a similar role, we first expressed and purified an NTD-deletion mutant (Δ1-66; ΔN MCP3) (Figure 4A). Due to the robust nature of MCP3 editing, single-turnover deacylation assays conducted at 27°C, which is the physiological temperature for growth of *Tb* in the procyclic form, were unable to detect differences in activity between wild-type (WT) and ΔN MCP3 (data not shown). We therefore monitored Ala-tRNA^Pro^ hydrolysis at 13.5°C and again observed little differences between the enzymes under single-turnover conditions (Supplementary Figure S2). We next conducted multiple-turnover steady-state assays at 27°C. Under these conditions, WT MCP3 hydrolyzed Ala-tRNA^Pro^ at an initial rate of ~1.2 nM/s while ΔN MCP3 displayed a rate of ~0.83 nM/s (Figure 4B). The 1.4-fold decrease in the initial velocity suggests that the NTD of MCP3 plays only a minor role in catalysis.

To determine the oligomeric state of MCP3 and the potential role of the NTD in dimerization, we performed SEC-MALS analyses with WT and ΔN MCP3. The WT protein consistently eluted as a dimer, evident from the ~60 kDa light-scattering peak (Figure 4D, pink trace). NTD deletion resulted in a loss of homodimerization, as ΔN MCP3 produced a light-scattering signal corresponding to a molar mass of ~23 kDa (Figure 4D; blue trace). We next used ITC to determine the strength of MCP3 homodimerization. A concentrated sample of WT MCP3 (250 μM) was titrated into a microcalorimeter cell containing buffer to measure the heat of dissociation of dimers into monomers; ITC determined a dimer dissociation constant (*K*_Dimer_) of ~2.6 μM (Figure 4C).

**Figure 5:**
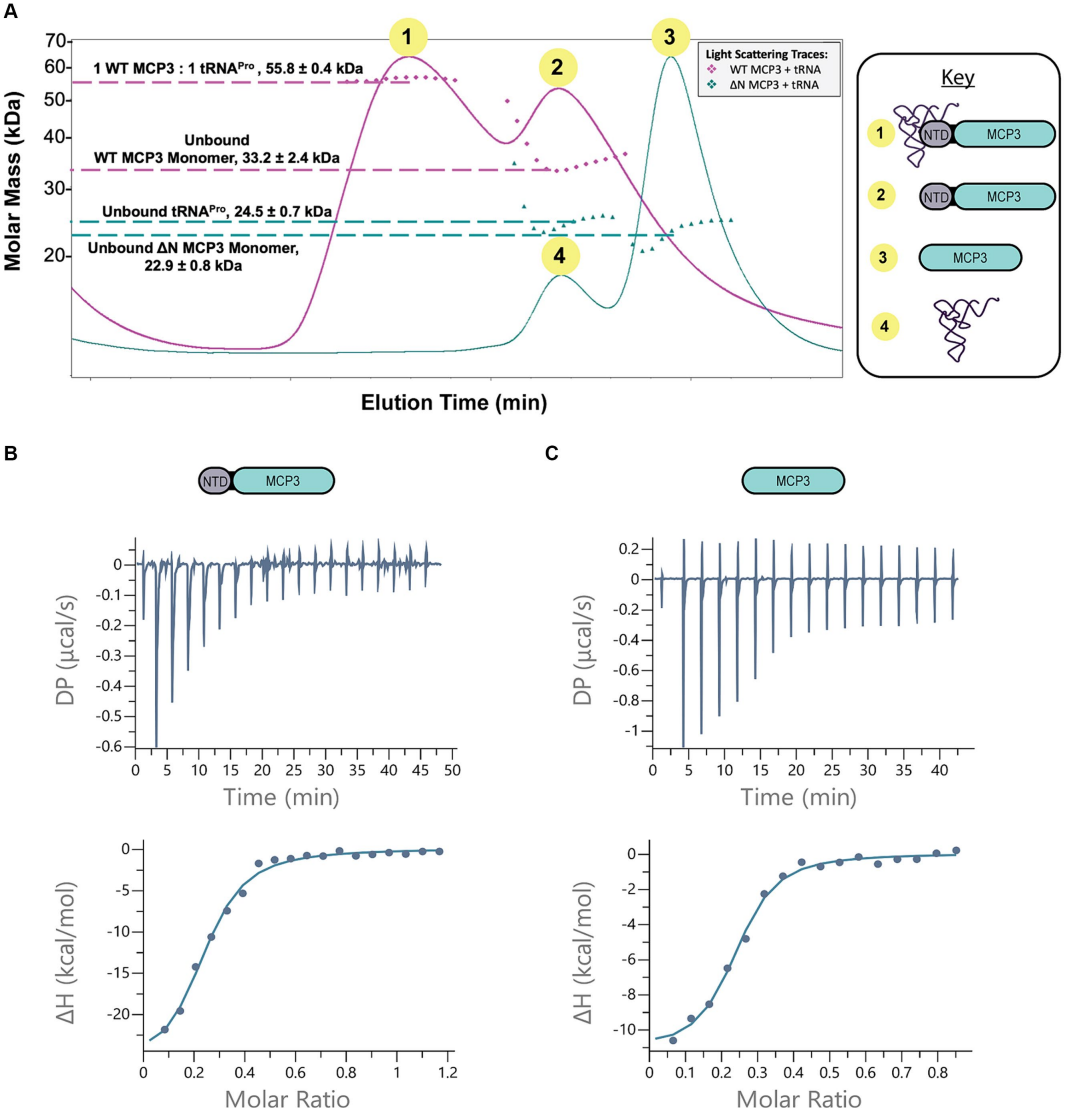
MCP3 NTD plays minor role in tRNA^Pro^ binding. **(A)** SEC-MALS analysis of WT (pink trace) and ∆N MCP3 (blue trace) in the presence of tRNA^Pro^. The tRNA was pre-incubated with 2.5-fold molar excess of MCP3 at 27°C prior to SEC-MALS analysis at 4°C. Annotated molar masses represent an average of three trials with standard deviation indicated. **(B,C)** ITC analysis of binding between WT MCP3 **(B)** or ∆N MCP3 **(C)** and tRNA^Pro^ at 25°C. Average K_D_ values and thermodynamic parameters are summarized in Table 3.

To determine the stoichiometry of tRNA^Pro^ binding to MCP3, we pre-incubated tRNA^Pro^ with 2.5-fold excess of WT MCP3 at 27°C and then analyzed complex formation by SEC-MALS at 4°C. We observed two light-scattering peaks, which suggested that WT MCP3 bound to tRNA^Pro^ as a monomer with 1:1 stoichiometry (~56 kDa peak 1), while at least some of the excess MCP3 eluted as a monomer rather than the expected dimer (Figure 5A, peak 2). We cannot rule out whether free MCP3 dimer co-eluted with the 1:1 MCP3:tRNA complex (Figure 5A, peak 1). For reference, the elution profile of tRNA^Pro^ alone is shown in Figure 4D (brown trace). Binding of tRNA^Pro^ consistently resulted in the dissociation of the MCP3 dimer in three independent trials. We repeated the experiment with ΔN MCP3 and observed no complex formation; tRNA^Pro^ and ΔN MCP3 eluted as two separate peaks (Figure 5A, peaks 3 and 4). As robust Ala-tRNA^Pro^ hydrolysis by ΔN MCP3 was observed (Figure 4B; Supplementary Figure S2), we know that the truncated protein can bind to aminoacyl-tRNA^Pro^. Therefore, we speculate that the aminoacyl moiety is required for stable tRNA binding by the truncated protein.

**Table 3:** Summary of thermodynamic parameters of WT and ΔN MCP3 binding to tRNA measured by ITC.^†^

	K_D_ (μM)	ΔH (kcal/mol)	−TΔS (kcal/mol)	ΔG (kcal/mol)
WT MCP3: tRNA^Pro^	1.05 ± 0.348	−24.3 ± 2.80	16.2 ± 2.95	−8.20 ± 0.283
ΔN MCP3: tRNA^Pro^	0.809 ± 2.33	−7.98 ± 4.70	−3.55 ± 4.50	−8.33 ± 0.177
WT MCP3: tRNA^Ala^	1.12 ± 0.300	−4.70 ± 0.540	−3.88 ± 0.790	−8.6 ± 0.309

^†^Values represent the average of three independent trials with standard deviations indicated.

To further explore the mechanism by which the NTD improves tRNA binding, we conducted ITC. The measured K_D_ of tRNA^Pro^ binding by WT MCP3 is ~1.0 μM; binding is enthalpically driven and entropically unfavorable (ΔH = −24 kcal/mol; −TΔS = +16.2 kcal/mol) (Figure 5B and Table 3). ΔN MCP3 binds to tRNA^Pro^ with similar affinity (K_D_ = ~0.8 μM), but with lower enthalpy (ΔH = −7.98 kcal/mol) and a more favorable entropy (−TΔS = −3.55 kcal/mol) (Figure 5C and Table 3). The overall free energy of the interaction of ΔN and WT MCP3 with tRNA^Pro^ is nearly identical (ΔG_WT_ = −8.20 kcal/mol; ΔG_ΔN_ = −8.33 kcal/mol), which suggests the MCP3 NTD is not critical for tRNA binding.

### 3.4 FL ProRS depends on the discriminator base while the context of the discriminator base is important for tRNA deacylation by MCP3

*Cc* and *Hs* ProXp-ala depend on the first base-pair (N1:N72) and discriminator base (N73) of tRNA^Pro^ for recognition (Vargas-Rodriguez et al., 2020; Ma et al., 2023) and both editing domains evolved to recognize these tRNA^Pro^ identity elements in a species-specific manner—bacterial type tRNA^Pro^ (C1:G72, A73) is a poor substrate for *Hs* ProXp-ala and vice versa. Since characterization of the tRNA identity elements of ProXp-ala domains has primarily been conducted with stand-alone enzymes, we hypothesized the appendage of ProXp-ala to the ProRS catalytic domain would eliminate the dependence on acceptor stem identity elements for FL *Tb* ProRS. Indeed, previous characterization of *E. coli* ProRS showed that editing by the INS domain was independent of changes in the acceptor stem (Das et al., 2014). Similarly, *P. falciparum* ProRS, which has an N-terminal ProXp-ala domain like *Tb* ProRS, was previously reported to hydrolyze *E. coli* Ala-tRNA^Pro^ (C1:G72, A73), although activity with cognate *P. falciparum* Ala-tRNA^Pro^ (C1:G72, C73) was not tested (Ahel et al., 2003). Conversely, the free-standing MCP3 protein may depend more strongly on tRNA acceptor stem elements.

**Figure 6:**
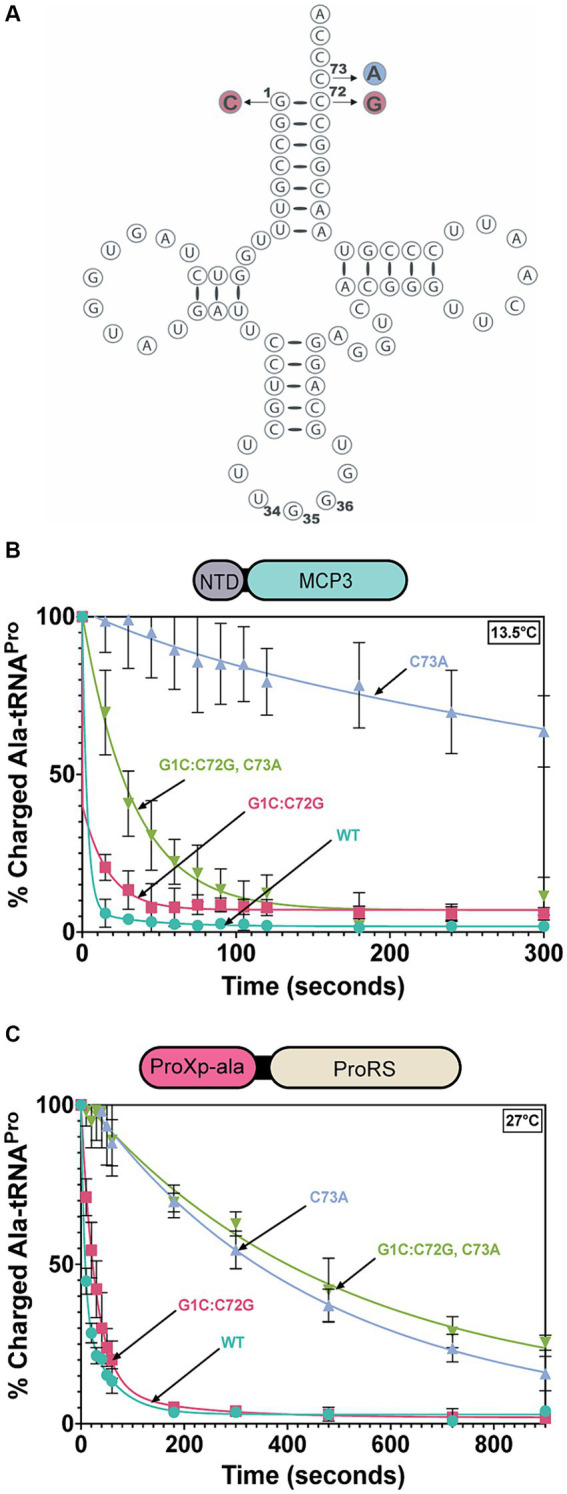
Acceptor stem specificity of MCP3 and ProRS. **(A)** Cloverleaf model of Tb tRNA^Pro^_(UGG)_ with mutations indicated: G1C:C72G (red), C73A (blue). **(B)** Single-turnover deacylation of WT (teal), G1C:C72G (red), C73A (grey), and G1C:C72G, C73A (green) Ala-tRNA^Pro^ by MCP3 at 13.5°C. **(C)** Single-turnover deacylation of WT (teal), G1C:C72G (red), C73A (grey), and G1C:C72G, C73A (green) Ala-tRNA^Pro^ by FL ProRS at 27°C. Error bars represent the average of three independent trials. Rate constants are summarized in Table 4.

Tb tRNA^Pro^ contains acceptor stem elements G1:C72 and C73, similar to other eukaryotic tRNA^Pro^ isoacceptors (Chan and Lowe, 2016). To test the tRNA^Pro^ acceptor stem specificity of *Tb* MCP3 and FL ProRS, we transcribed and mis-alanylated three acceptor stem variants: C73A, G1C:C72G, and G1C:C72G, C73A (Figure 6A). Introduction of these mutations mimics the tRNA^Pro^ identity elements encoded by bacteria. At 13.5°C, all three mutations perturb the activity of MCP3 to varying extents (Figure 6B and Table 4). The *k*_obs_ for MCP3 hydrolysis of C73A Ala-tRNA^Pro^ was reduced ~28-fold relative to hydrolysis of WT Ala-tRNA^Pro^. Transversion of the first base pair only impacted the *k*_obs_ ~ 1.5-fold. Since the triple mutant contained the C73A mutation, we predicted it would be the poorest substrate for MCP3. Interestingly, when coupled with first base pair transversion, MCP3 is not as reliant on the discriminator base with a modest 6.4-fold decrease in *k*_obs_. Thus, the context of the base at position 73 appears to be important for deacylation by MCP3. FL ProRS *trans*-editing activity is sensitive to the identity of the discriminator base independent of context—introduction of C73A and all three acceptor stem mutations (triple mutant) reduced the rate constant of Ala-tRNA^Pro^ deacylation by ~35-fold (Figure 6C and Table 4). FL *Tb* ProRS was also relatively insensitive to 1:72 base pair transversion as the *k*_obs_ value was only reduced ~2-fold. Therefore, our observation that FL ProRS strongly depends on acceptor elements differs from the results reported for *E. coli* ProRS (Ahel et al., 2003; Das et al., 2014). *P. falciparum* ProRS may also depend on acceptor stem elements as *E. coli* Ala-tRNA^Ala^ was not deacylated by this enzyme, although whether this apparent tRNA specificity was due to acceptor stem differences between *E. coli* tRNA^Pro^ and tRNA^Ala^ was not determined (Ahel et al., 2003; Das et al., 2014).

**Table 4:** Single-turnover rate constants for deacylation of tRNA^Pro^ acceptor-stem mutants mischarged with alanine by MCP3 and FL ProRS.

Ala-tRNA^Pro^ Acceptor Stem Mutant	MCP3 *k*_obs_ (sec^−1^)*	Fold-change^†^	FL ProRS *k*_obs_ (sec^−1^)*	Fold-change^†^
WT	0.198 ± 0.068	–	0.069 ± 0.010	–
C73A	0.007 ± 0.006	28	0.002 ± 0.0002	35
G1:C72G	0.127 ± 0.015	1.5	0.032 ± 0.009	2.2
G1:C72G, C73A	0.031 ± 0.008	6.4	0.002 ± 0.0003	35

*k_obs_ values determined using 500 nM enzyme and 50 nM Ala-tRNA^Pro^ at 27°C. Standard deviations represent the average of three independent trials. †Fold-change is based on the WT Ala-tRNA^Pro^ deacylation.

**Figure 7:**
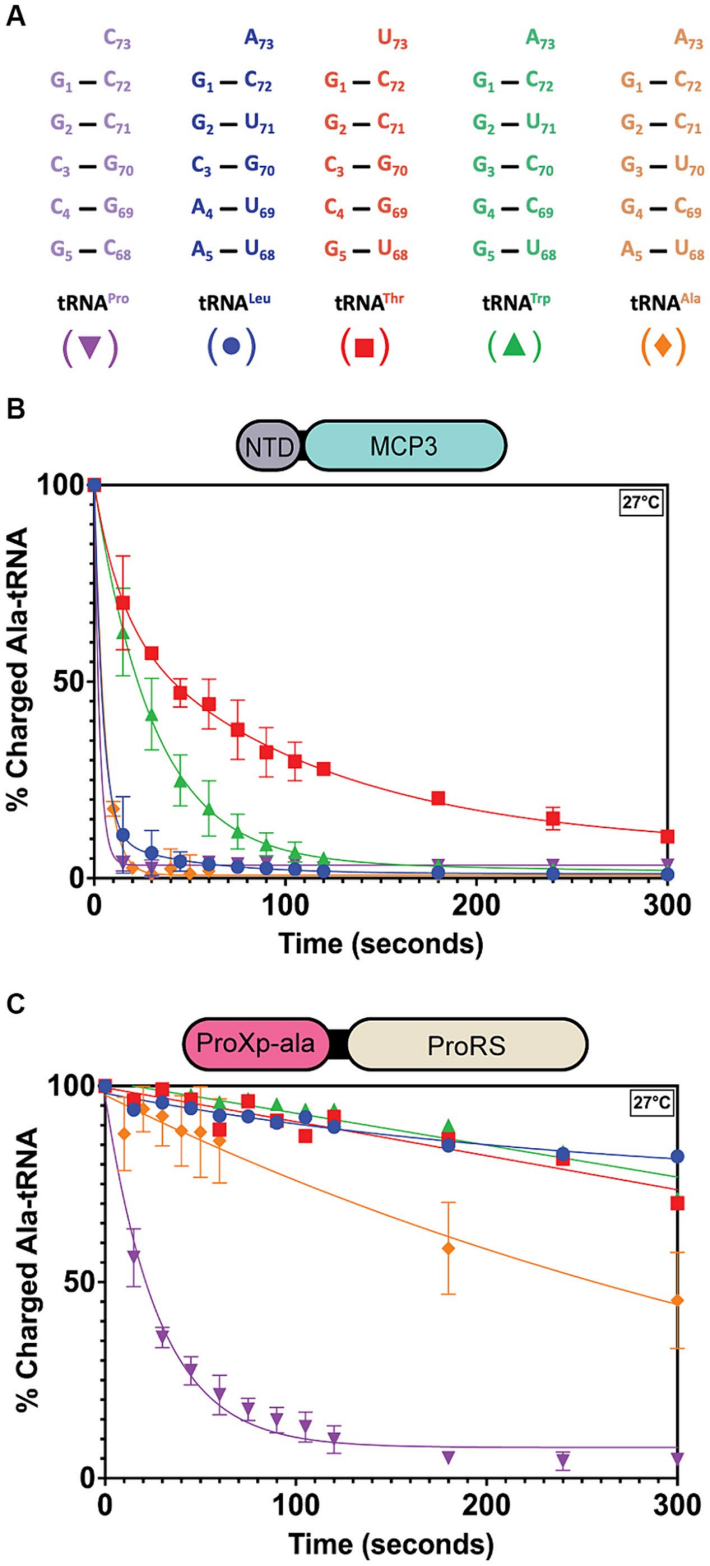
*Tb* MCP3 and ProRS deacylation of G1:C72-containing tRNAs with altered discriminator base. **(A)** Acceptor stem sequences of *Tb* tRNAs mis-charged with alanine and tested for deacylation. Colors and symbols match the deacylation curves in panels **(B,C)**. **(B)** Single-turnover deacylation of Ala-tRNAs by MCP3 at 27°C. Error bars represent an average of three trials. **(C)** Single-turnover deacylation of Ala-tRNAs by FL ProRS at 27°C. Error bars reflect the standard deviation between three trials. Results of deacylation of Ala-tRNA^Thr^, tRNA^Trp^, and tRNA^Leu^ by ProRS are based on a single trial. Rate constants are summarized in Table 5.

Since the context of the discriminator base appears to affect the tRNA specificity of MCP3, we wondered whether other tRNA species with G1:C72 base pairs but altered N73 bases, such as tRNA^Ala^, may be substrates for MCP3. To explore this idea, we *in vitro* transcribed four additional *Tb* tRNAs, mischarged them with alanine, and performed deacylation assays. Of the tRNAs selected, all contain a G1:C72 base pair (like tRNA^Pro^) and encode either A73 or U73 at the discriminator position (Figure 7A). At 27°C, MCP3 appeared to deacylate Ala-tRNA^Pro^, -tRNA^Ala^, and -tRNA^Leu^ with similar efficiency (Figure 7B and Table 5). Ala-tRNA^Thr^ and -tRNA^Trp^ showed slower deacylation by MCP3 but were still hydrolyzed to near completion within 300 s. In contrast, assays performed with FL ProRS showed significantly reduced Ala-tRNA deacylation activity for all tRNA species tested relative to Ala-tRNA^Pro^ (Figure 7C and Table 5). These results suggest that MCP3 is a promiscuous Ala-tRNA deacylase.

**Table 5:** Single-turnover rate constants for deacylation of various tRNAs mischarged with alanine by MCP3.

Ala-tRNA^i^	MCP3 *k*_obs_ (sec^−1^)*	FL ProRS *k*_obs_ (sec^−1^)^⧫^
Pro	0.329 ± 0.056	0.030
Ala	0.310 ± 0.080	0.002
Leu	0.180 ± 0.069	ND
Thr	0.018 ± 0.005	ND
Trp	0.032 ± 0.007	ND

*k_obs_ values determined using 500 nM enzyme and 50 nM Ala-tRNA at 27°C. Standard deviations represent the average of three independent trials. ND, not detected. ^⧫^*k*_obs_ reported for FL is a single trial (*n* = 1) determined using 500 nM enzyme and 50 nM Ala-tRNA at 27°C. ^ℹ^Amino acid in first column refers to the identity of the tRNA acceptor.

### 3.5 MCP3 deacylation of Ala-tRNA^Ala^

**Figure 8:**
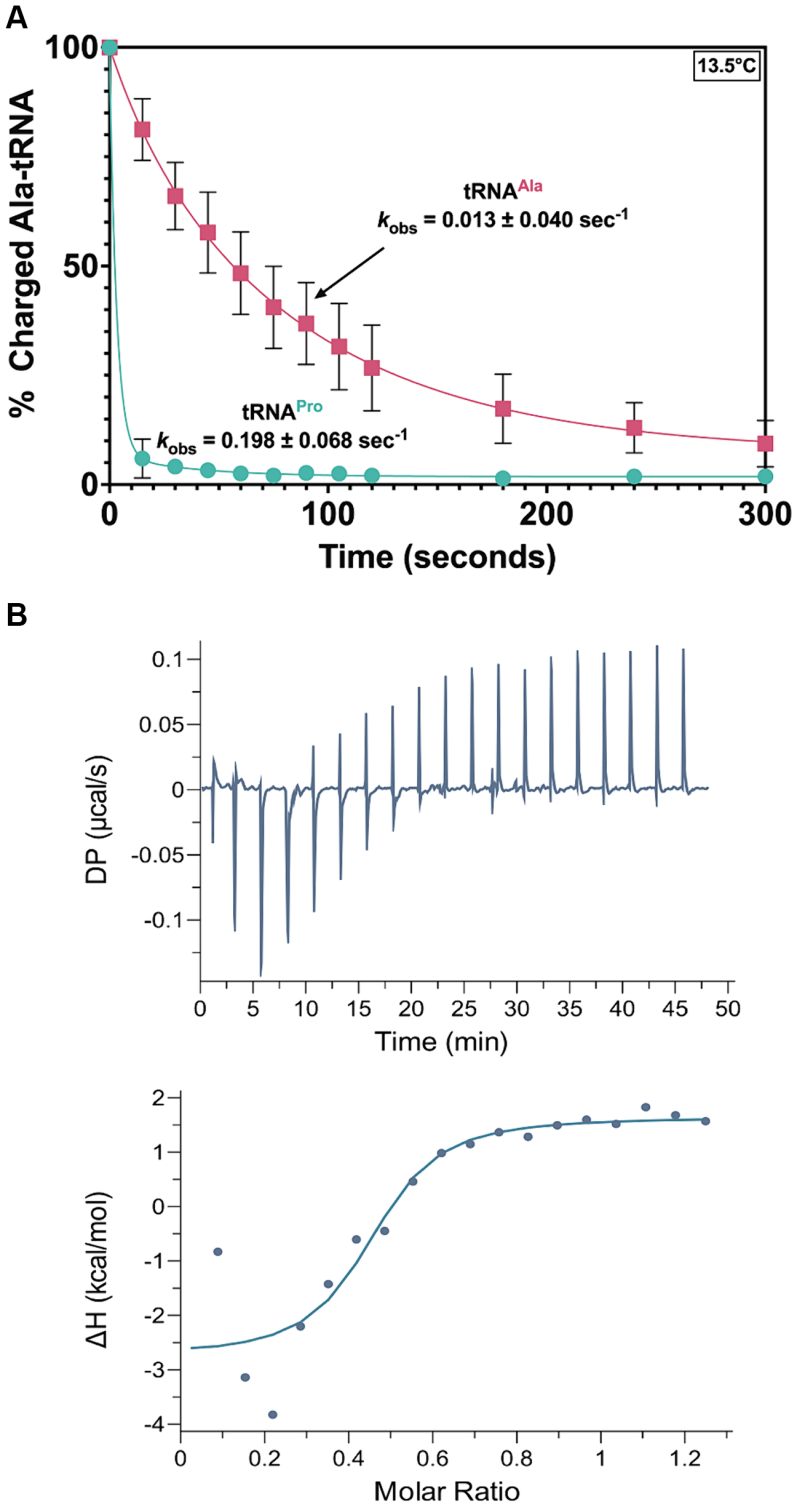
MCP3 deacylation and interaction with Ala-tRNA^Ala^. **(A)** Deacylation of Ala-tRNA^Ala^ (red ■) and Ala-tRNA^Pro^ (teal ●) by MCP3 at 13.5°C. Error bars indicate the average of three independent trials. **(B)** ITC analysis of MCP3 binding to tRNA^Ala^ at 25°C. Average K_D_ values and thermodynamic parameters are summarized in Table 3.

Promiscuous deacylation of cognate Ala-tRNA^Ala^ would be problematic for *T. brucei* fitness. Our previous deacylation assays were conducted at 27°C to mimic physiological conditions, but at this temperature, deacylation was so rapid that it was difficult to detect differences among Ala-tRNA^Pro^ and -tRNA^Ala^. Therefore, we conducted deacylation assays at 13.5°C; under these conditions, a ~16-fold reduction in the *k*_obs_ for Ala-tRNA^Ala^ was measured relative to Ala-tRNA^Pro^ (Figure 8A). We hypothesize the lower temperature reduces dynamic motion of the NTD and side chains within the catalytic domain of MCP3, which results in less flexibility in accommodating tRNAs that diverge in sequence from tRNA^Pro^. This suggests MCP3 may interact with tRNAs using different binding modes.

To test this idea, we used ITC to determine the thermodynamic parameters for tRNA^Ala^ binding by MCP3. The K_D_ (1.12 μM) and overall free energy (ΔG = −8.6 kcal/mol) of tRNA^Ala^ binding by MCP3 were nearly identical to the values for tRNA^Pro^ (Figure 8B and Table 3). However, WT MCP3 bound to tRNA^Pro^ in a predominantly enthalpic manner, with an unfavorable entropic cost—this is not observed for tRNA^Ala^ binding. WT MCP3 bound to tRNA^Ala^ in a manner that resembled ΔN MCP3:tRNA^Pro^ binding—a combination of favorable hydrogen bonding (ΔH ≈ −5 kcal/mol) and hydrophobic effects (−TΔS ≈ −4 kcal/mol) (Table 3).

### 3.6 Ala-tRNA^Ala^ protection by *Tb* MSC members

**Figure 9:**
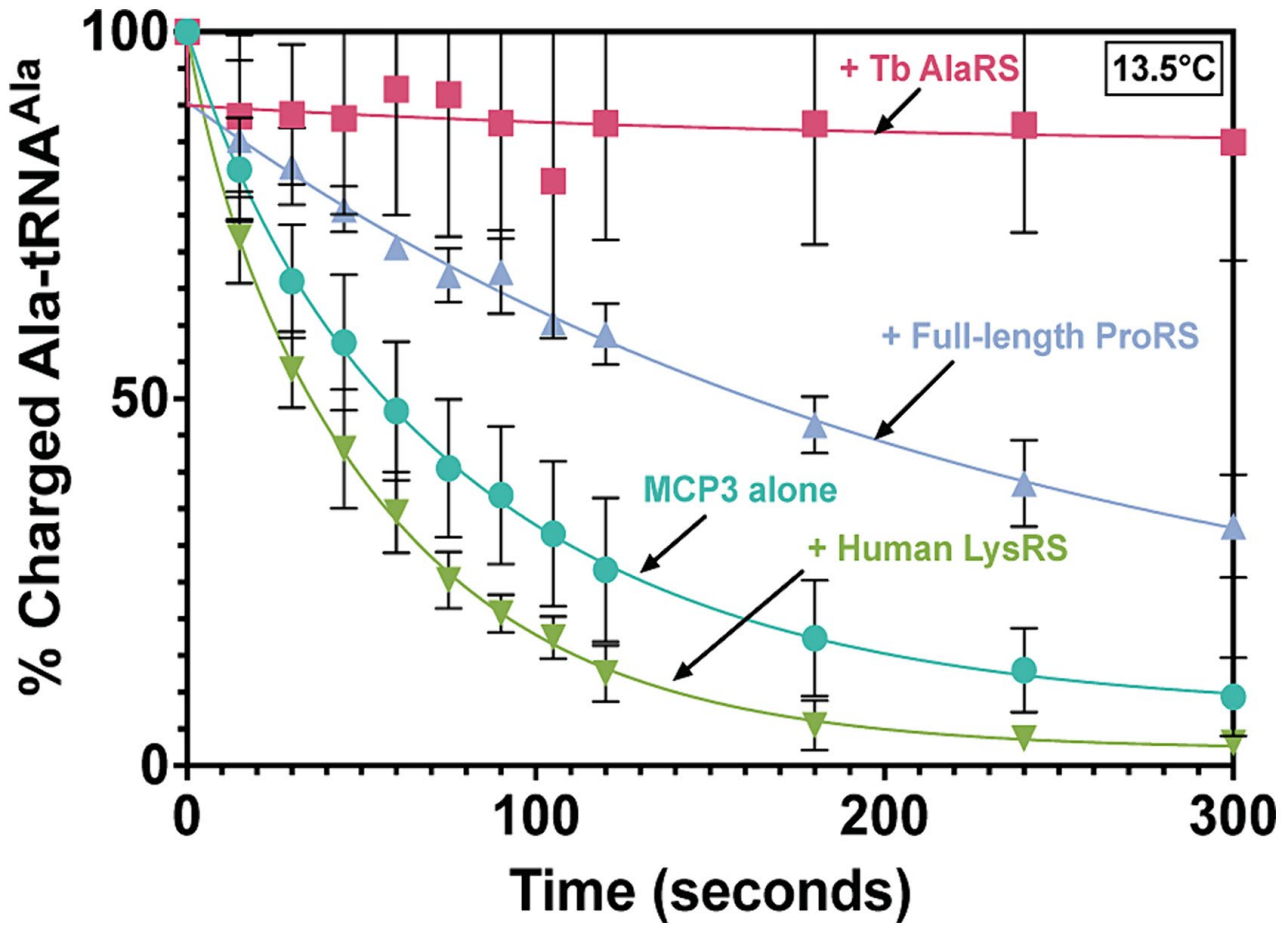
Ala-tRNA^Ala^ deacylation by MCP3 in the absence and presence of ARSs. Single-turnover deacylation of Ala-tRNA^Ala^ by MCP3 alone (teal●) or by MCP3 in the presence of *Tb* AlaRS (red ■), ProRS (grey▲), or *Hs* LysRS (green ▼) at 13.5°C. Proteins were at a final concentration of 500 nM. Error bars indicate average of three independent trials. Rate constants are summarized in Table 6.

MCP3 is a robust, promiscuous deacylase of cognate Ala-tRNA^Ala^ at 27°C. Our previous kinetic assays lacked protein–protein interactions that could potentially regulate the activity of MCP3. Since MCP3 is predicted to assemble with the *Tb* MSC, we performed Ala-tRNA^Ala^ deacylation assays in the presence of *Tb* AlaRS and ProRS. Before deacylation assays, 500 nM MCP3 was pre-incubated with an equimolar amount of FL *Tb* ProRS, *Tb* AlaRS, or *Hs* LysRS (as a negative control) at 27°C in a buffer also containing 0.1 mg/mL bovine serum albumin. In the presence of *Hs* LysRS, we observed no decrease in Ala-tRNA^Ala^ hydrolysis by MCP3 (Figure 9). Pre-incubation with FL ProRS reduced the single-turnover rate constant ~2-fold, while AlaRS completely protected Ala-tRNA^Ala^ from deacylation by MCP3 at 13.5°C (Figure 9). Significant protection by AlaRS is also observed 27°C (Supplementary Figure S3).

**Table 6:** Single-turnover rate constants of Ala-tRNA^Ala^ protection assays with MCP3.

Ala-tRNA^Ala^ Protection	MCP3 *k*_obs_ (sec^−1^)*	Fold-change^†^
MCP3 alone	0.013 ± 0.004	–
MCP3 + FL ProRS	0.007 ± 0.002	1.9
MCP3 + AlaRS	ND	>30
MCP3 + Hs LysRS	0.019 ± 0.004	+1.5

*k_obs_ values determined using 500 nM enzyme, 500 nM ARS, and 50 nM Ala-tRNA^Ala^ at 13.5°C. Standard deviations represent the average of three independent trials. ND, not detected. †Fold-change is based on MCP3 activity.

## 4 Discussion

In this work, we provide evidence for robust Ala-tRNA^Pro^ proofreading mechanisms encoded by *T. brucei*, which likely evolved out of a need to maintain translational fidelity amidst an alanine-rich amino acid pool. Of all metabolites in the parasite, alanine has been reported to be one of the most abundant (Creek et al., 2015; Smith et al., 2017; Johnston et al., 2019). The amino acid pool is enriched with alanine following proline catabolism in the insect-stage, transamination of pyruvate produced from glycolysis in the bloodstream parasite, and through direct import from the extracellular amino acid pool (Mantilla et al., 2017; Haindrich et al., 2021). We showed that both the ProXp-ala domain appended to ProRS and MCP3 possess robust Ala-tRNA^Pro^ deacylase activity *in vitro*. Whether the ProXp-ala domain of *Tb* ProRS and/or MCP3 are required for proofreading within the parasite remain open questions.

The essentiality of the proline-rich procyclin coat (Ruepp et al., 1997) in the insect is the likely reason for the requirement for redundant and robust Ala-tRNA^Pro^ editing. According to the TriTrypDB Kinetoplastid database (Alvarez-Jarreta et al., 2024) a shotgun RNAi screen of protein-coding genes identified the MCP3 gene as essential for parasite fitness (Alsford et al., 2011). MCP3 essentiality will be explored further in the future. Our data suggest that the fusion of ProXp-ala to ProRS resulted in improved deacylation activity relative to a free ProXp-ala, which is encoded in many species. Related protozoans to *Tb*, such as *T. cruzi* and *Leishmania*, also encode both a ProRS-fused ProXp-ala and MCP3 and possess large alanine pools (Simon et al., 1983). Although fused to ProXp-ala, we expect ProRS to exhibit fast product release as a class II synthetase (Zhang et al., 2006) and edit mischarged tRNA predominantly in *trans*.

Not all organisms that encode an MCP3-like factor catabolize proline or have abundant alanine pools. For example, *A. thaliana*, which encodes a free (non-MSC) MCP3 domain and a stand-alone ProXp-ala domain housed in the *At* MSC (McWhite et al., 2020), does not appear to have a significant alanine pool (Watanabe et al., 2013; Hildebrandt et al., 2015). Therefore, one could speculate that ProRS mischarging events are less frequent in plants than in *Tb*, and/or MCP3 may be localized to non-cytoplasmic compartments for tRNA^Pro^ proofreading or other non-canonical functions. Alternatively, MCP3 may have evolved to maintain homeostasis in organisms that are forced to endure environmental stress conditions—*T. brucei* are dependent on the host for survival and must endure the same stress as the host. Plants are similar in that they are sessile and rely on their immediate surroundings, wherein nutrient availability and predation is often dynamic. In contrast, MCP3 is not encoded by highly autonomous organisms (i.e., humans) that can readily adapt to stress conditions.

Whether assembly of both ProRS and MCP3 into the *Tb* MSC is an evolutionary coincidence, or a strategy used by the parasite to promote tRNA^Pro^ selectivity and avoid promiscuous deacylation is another question addressed by this work. In the absence of other factors, MCP3 robustly deacylated Ala-tRNA^Ala^; this activity was significantly diminished in the presence of AlaRS and to some extent by ProRS. tRNA promiscuity by an INS superfamily member is not unprecedented—previous characterization of *E. coli* YbaK revealed it lacks tRNA discrimination and selects its substrate through the amino acid moiety of a cysteinylated tRNA (An and Musier-Forsyth, 2005). As a result, Cys-tRNA^Cys^ proofreading is observed *in vitro*. However, cross-linking and mass spectrometry studies showed ternary complex formation between YbaK, ProRS, and tRNA^Pro^, suggesting YbaK gains specificity for tRNA^Pro^ through ProRS-mediated interactions (An and Musier-Forsyth, 2005; Chen et al., 2019).

The MCP3 NTD played a marginal role in tRNA affinity and catalysis but is essential for homodimerization. We speculate that MCP3 evolved the NTD to facilitate its interactions with the MSC, but this remains to be tested. A similar hypothesis was proposed for the role of the *At* ProXp-ala CTD in MSC assembly (Byun et al., 2022). The apparent shift of the dimeric *Tb* MCP3 protein to a monomeric species in the presence of tRNA (i.e., 1:1 binding stoichiometry) supports this conclusion. We observed robust catalysis from the ΔN enzyme yet failed to observe stable tRNA complex formation during SEC-MALS possibly due to the transient nature of the interaction in the absence of an aminoacyl moiety. Our ITC experiments revealed highly favorable hydrogen bonding (ΔH = −24.3 kcal/mol) between WT MCP3 and tRNA^Pro^, whereas the enthalpy of ΔN MCP3:tRNA^Pro^ binding, although still favorable, was lower in magnitude (ΔH = −7.98 kcal/mol). Thus, the NTD is making favorable hydrogen bonding contacts with tRNA^Pro^, which could help to maintain the integrity of the MCP3:tRNA complex during SEC-MALS.

The robust nature of MCP3 Ala-tRNA^Pro^ deacylation activity and its ability to accommodate different species of tRNA is likely due to unique residues absent in other INS superfamily members. For example, R80 in *Cc* ProXp-ala has been shown to be important for discriminator base recognition—an R80A or R80N mutation significantly impacts hydrolysis of A73-containing Ala-tRNA^Pro^ (Danhart et al., 2017; Ma et al., 2023). Residue N173 in *Tb* MCP3 and N86 in *Hs* ProXp-ala, which also displays a preference for C73 in tRNA^Pro^, align with R80 in *Cc* ProXp-ala (Vargas-Rodriguez et al., 2020). R80N *Cc* ProXp-ala has been shown to deacylate C73-containing Ala-tRNA^Pro^ (Ma et al., 2023). Thus, we postulate N173 in *Tb* MCP3 interacts with the discriminator base of WT *Tb* tRNA^Pro^. However, the mechanism used by MCP3 to adapt to changes in the discriminator base needs further exploration.

Taken together, our initial biochemical characterization of *Tb* MCP3 informs future studies of conserved residues among MCP3-homologs that participate in tRNA binding and catalysis. The AlphaFold2 model of the *Tb* MCP3 catalytic domain shows it is structurally similar to ProXp-ala. In the absence of other factors, MCP3 is a strong deacylase of both cognate Ala-tRNA^Ala^ and Ala-tRNA^Pro^. Our current findings suggest one role of the *Tb* MSC is to ensure alanine codon fidelity through sequestration of MCP3. Specifically, we postulate the assembly of MCP3, AlaRS, and ProRS in the same complex gatekeeps correctly aminoacylated tRNAs from MCP3 and prevents competition with elongation factor 1-alpha (eEF1-⍺), which binds and delivers all aminoacyl-tRNA species to the ribosome. Previous characterization of the *Tb* MSC supports this hypothesis as AlaRS and ProRS were not found to directly interact with MCP3 (Cestari et al., 2013). Other factors that could offer tRNA^Ala^ protection from MCP3 are tRNA modifications and the presence of other modified tRNA species. An open question in the field of *trans*-editing factors is whether tRNA modifications act as anti-determinants for proofreading (Giegé and Eriani, 2023; Yared et al., 2024). Future work will determine the impact of the *Tb* MCP3 interactome and the role of post-transcriptional/translational modifications on the activity of this editing domain.

## Data availability statement

All data described are contained within the article. Requests to access the datasets should be directed to KM-F musier-forsyth.1@osu.edu.

## Author contributions

RW: Conceptualization, Writing – review & editing, Data curation, Formal analysis, Investigation, Methodology, Validation, Visualization, Writing – original draft. AV: Data curation, Investigation, Methodology, Writing – review & editing. IS: Data curation, Writing – review & editing. KM-F: Conceptualization, Funding acquisition, Project administration, Resources, Supervision, Writing – review & editing.
