# Supplementary material for: Trypanosoma brucei multi-aminoacyl-tRNA synthetase complex formation limits promiscuous tRNA proofreading
Source: Front Microbiol. 2024 Jul 16;15:1445687. doi: 10.3389/fmicb.2024.1445687 (PMC11286415; doi:10.3389/fmicb.2024.1445687)
Supplement: Supplementary file 1 [file Data_Sheet_1.docx]

Supplementary Material

*Trypanosoma brucei* multi-aminoacyl-tRNA synthetase complex formation limits promiscuous tRNA proofreading

Rylan Watkins, Anna Vradi, Irina Shulgina, Karin Musier-Forsyth*

Department of Chemistry and Biochemistry, Center for RNA Biology, Ohio State University, Columbus, OH, USA

*** Correspondence:** Karin Musier-Forsyth; musier-forsyth.1@osu.edu

# Supplementary Figures and Tables

## Supplementary Figures

*
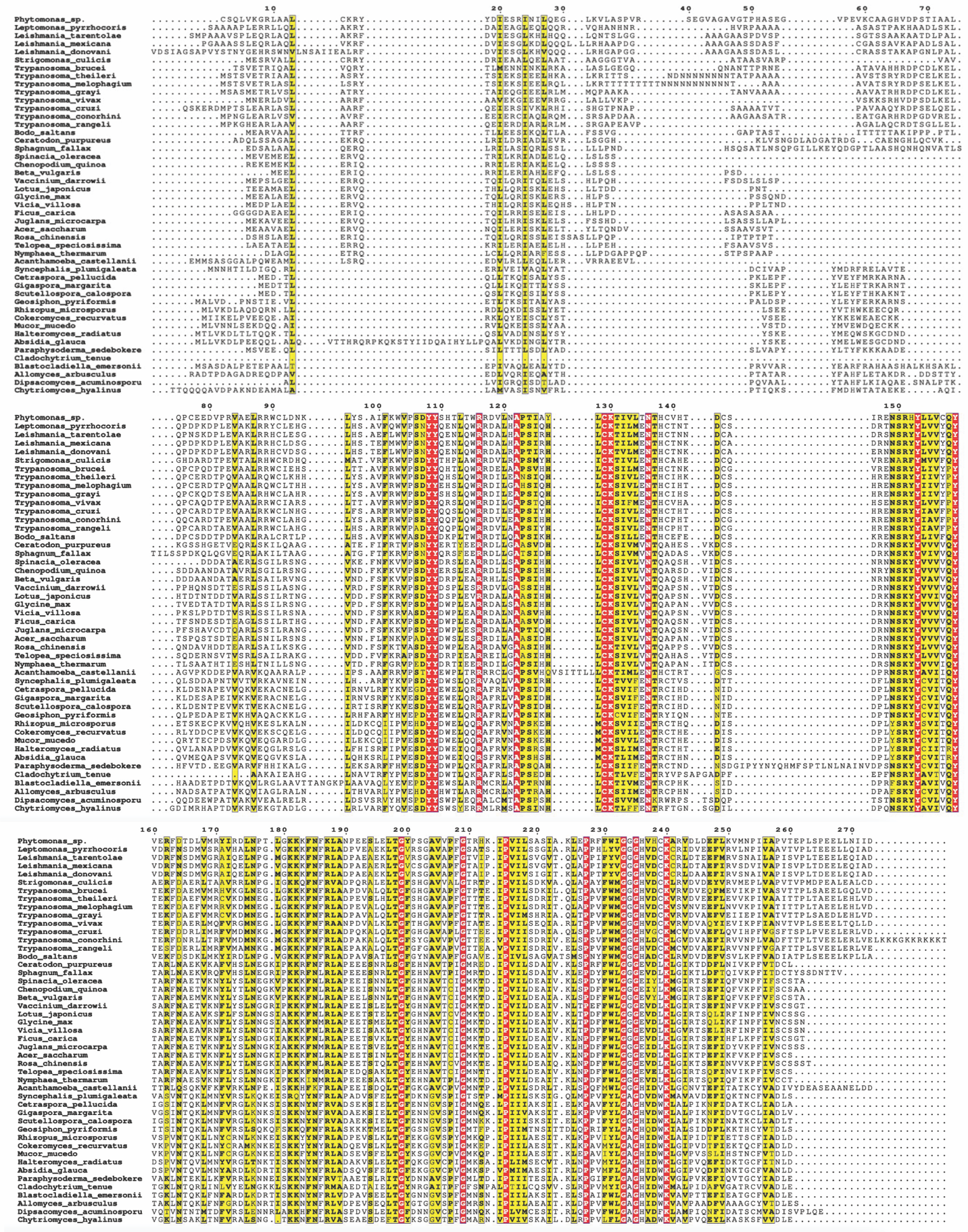
*

**Supplemental Figure 1. Multiple sequence alignment of MCP3-like proteins encoded by plants, protists, and fungi**. Highly conserved residues are highlighted in red whereas similar residues in yellow.


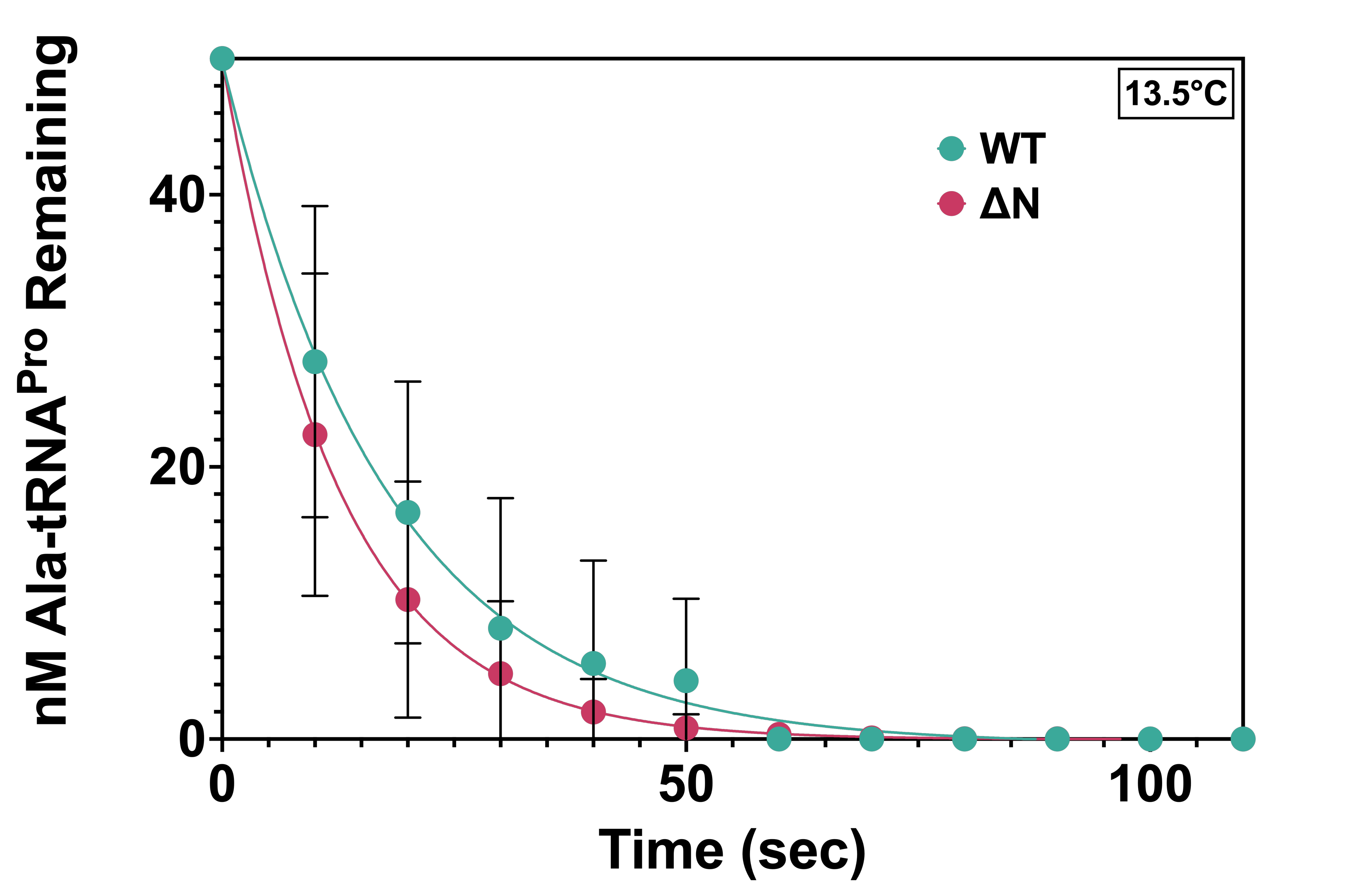


**Supplemental Figure 2. Single-turnover deacylation of Ala-tRNA^Pro^ by WT MCP3 and ΔN MCP3 at 13.5°C.** Deacylation of 50 nM Ala-tRNA^Pro^ with 500 nM of WT or ΔN MCP3. Error bars represent the average of two independent trials


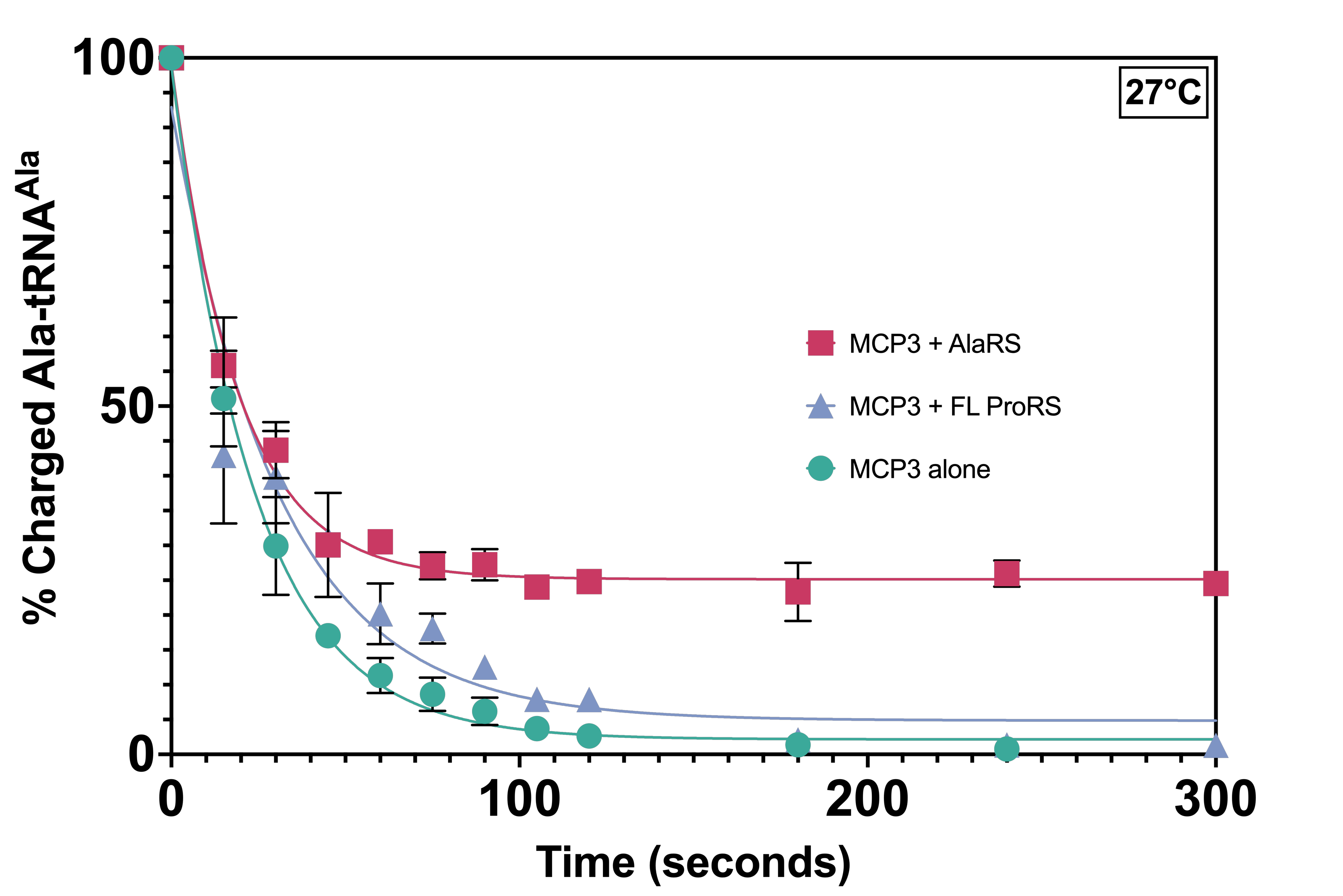


**Supplemental Figure 3. Ala-tRNA^Ala^ deacylation by MCP3 in the absence and presence of *Tb* AlaRS and ProRS at 27°C.** Single-turnover deacylation of 50 nM Ala-tRNA^Ala^ by 500 nM MCP3 alone (teal; ●) or in the presence of equimolar AlaRS (red; ■) or ProRS (grey; ▲).

**1.2 Supplementary Tables**

| **Protein** | **Isolation Buffer (lysis/equilibration/wash)** | **Storage Buffer** |
| --- | --- | --- |
| *Tb* 6xHis MCP3 | 100 mM Tris-HCl pH 8.0  600 mM NaCl  5% glycerol  20 mM β-mercaptoethanol  5 mM imidazole | 50 mM Tris-HCl pH 8.0  200 mM NaCl  2 mM dithiothreitol (DTT)  40% v/v glycerol |
| *Tb* 6xHis-GST-AlaRS | 100 mM MOPS-KOH pH 7.8  1 M NaCl  15% w/v sucrose  0.5% w/v CHAPS  20 mM β-mercaptoethanol  5 mM imidazole | 50 mM MOPS-KOH pH 7.8  500 mM NaCl  2 mM DTT  40% v/v glycerol |
| *Tb* 6xHis ΔProXp-ala ProRS | 50 mM NaPi pH 7.5  600 mM NaCl  10% glycerol  20 mM β-mercaptoethanol  5 mM imidazole | 50 mM Tris-HCl pH 8.0  500 mM NaCl  5 mM DTT  40% v/v glycerol |
| *Tb* 6xHis Full-length ProRS | 100 mM MOPS-KOH pH 7.8  1 M NaCl  50 mM L-Glu  50 mM L-Arg  15% glycerol  10 mM DTT  5 mM imidazole  1% w/v CHAPS | 25 mM MOPS-KOH pH 7.8  500 mM NaCl  5% D-trehalose  5 mM Tris-(2-carboxyethyl)-phosphine (TCEP)  40% v/v glycerol |

**Supplement Table 1.** Buffer used for isolation and storage of recombinant *T. brucei* proteins.

**
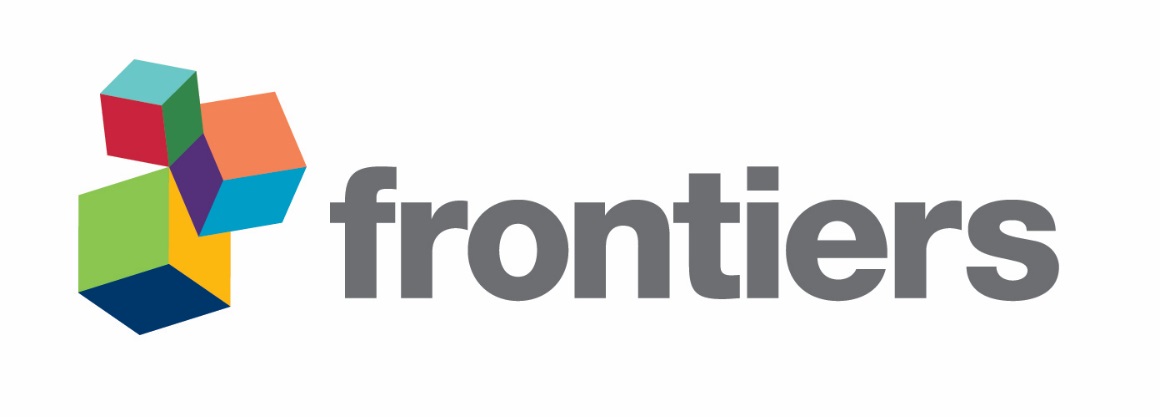
**

**Supplementary Figure 1.** The figure legends are required to have the same font as the main text, 12 point normal Times New Roman, single spaced. Please use a single paragraph for each legend and prepare the figures keeping in mind the PDF layout.
